# Non-Imidazole Histamine H_3_ Ligands. Part VII. Synthesis, In Vitro and In Vivo Characterization of 5-Substituted-2-thiazol-4-*n*-propylpiperazines

**DOI:** 10.3390/molecules23020326

**Published:** 2018-02-03

**Authors:** Roman Guryn, Marek Staszewski, Anna Stasiak, Daniel McNaught Flores, Wiesława Agnieszka Fogel, Rob Leurs, Krzysztof Walczyński

**Affiliations:** 1 Department of Synthesis and Technology of Drugs, Medical University of Lodz, ul. Muszyńskiego 1, 90-145 Łódź, Poland; roman.guryn@umed.lodz.pl (R.G.); marek.staszewski@umed.lodz.pl (M.S.); 2 Department of Hormone Biochemistry, Medical University of Lodz, ul. Żeligowskiego 7/9, 90-752 Łódź, Poland; anna.stasiak@umed.lodz.pl (A.S.); wieslawa.agnieszka.fogel@umed.lodz.pl (W.A.F.); 3 Amsterdam Institute of Molecules, Medicines & Systems, Division of Medicinal Chemistry, Vrije Universiteit Amsterdam, De Boelelaan 1108, 1081 HZ Amsterdam, The Netherlands; d.mcnaughtflores@vu.nl (D.M.F.); r.leurs@vu.nl (R.L.)

**Keywords:** histamine H_3_ receptor non-imidazole antagonists, *N*-methyl-*N*-ω-phenylalkyl-ω-[2-(4-n-propylpiperazin-1-yl)-1,3-thiazol-5-yl]alkan-1-amines

## Abstract

H_3_ receptors present on histaminergic and non-histaminergic neurons, act as autoreceptors or heteroreceptors controlling neurotransmitter release and synthesis. Previous, studies have found that the compound *N*-methyl-*N*-3-phenylalkyl-2-[2-(4-n-propylpiperazin-1-yl)-1,3-thiazol-5-yl]ethan-1-amine (**ADS-531, 2c**) exhibits high in vitro potency toward H_3_ guinea pig jejunal receptors, with pA_2_ = 8.27. To optimize the structure of the lead compound **ADS-531**, a series of 5-substituted-2-thiazol-4-*n*-propylpiperazines **3** were synthesized and subjected to in vitro pharmacological characterization; the alkyl chain between position 2 of the thiazole ring and the terminal secondary *N*-methylamino function was elongated from three to four methylene groups and the *N*-methylamino functionality was substituted by benzyl-, 2-phenylethyl-, and 3-phenyl-propyl- moieties. SAR studies on novel non-imidazole, 5-substituted-2-thiazol-4-*n*-propyl-piperazines **3** showed that the most active compound **3a** (pA_2_ = 8.38), additionally possessed a weak competitive H_1_-antagonistic activity. Therefore, compound **ADS-531**, which did not exhibit any H_1_-antagonistic activity, was chosen for further evaluation for its affinity to the recombinant rat and human histamine H_3_ receptors (rH_3_R and hH_3_R, respectively). **ADS-531** exhibited nanomolar affinity for both rH_3_R and hH_3_R receptors. It was also shown that, **ADS-531** given subchronically to rats (s.c. 3 mg/kg, 5 days) penetrated the brain, where it affected dopamine, noradrenaline and serotonin concentration; however, it did not affect histamine concentration nor feeding behavior.

## 1. Introduction

The H_3_ receptors mediate the diverse biological effects of the neurotransmitter histamine [1] and they are widely expressed in the mammalian brain, particularly in areas involved in cognitive processes and arousal, i.e., the cerebral cortex, hippocampus, basal ganglia, and hypothalamus [2,3]. H_3_ receptors are located on histaminergic or non-histaminergic neurons, respectively acting as autoreceptors or heteroreceptors, controlling the release and synthesis of histamine [4] and of multiple neurotransmitters such as acetylcholine [5], norepinephrine [6] and dopamine [7]. These data suggested that H_3_ antagonists could affect a number of behaviors and be useful in the treatment of cognitive deficits associated with a variety of disease states including Alzheimer’s disease (AD) [8], attention deficit hyperactivity disorder (ADHD) [9], schizophrenia [10], and obesity [11].

**Figure 1:**
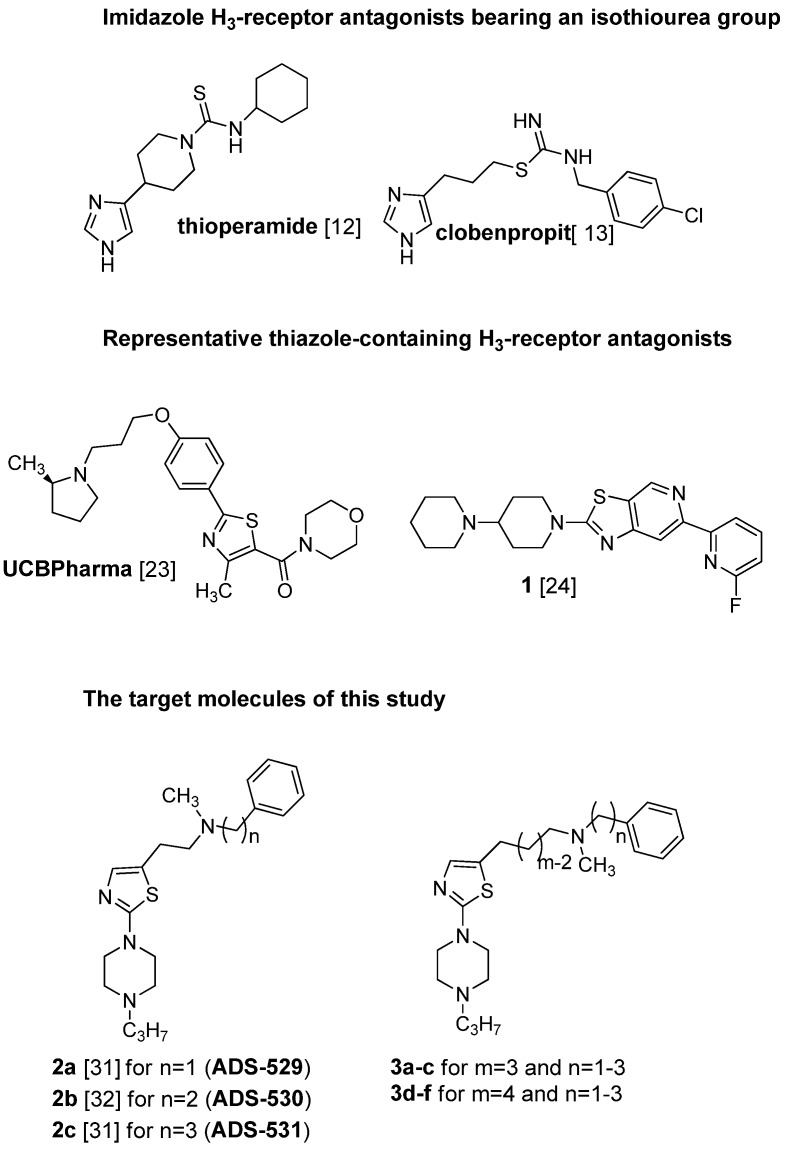
Structures of some known histamine H_3_-receptor antagonists and the target molecules of this study.

The first generation of active histamine H_3_ receptor antagonists were designed on the basis of a structural modification of the endogenous ligand, histamine, wherein the imidazole ring plays an important role [12]. Widely known representatives are thioperamide [13], and clobenpropit [14], both containing an isothiourea group (Figure 1).

Many ligands of this type have found utility in experimental studies as pharmacological tools [15]. However, the presence of an imidazole ring with strong hydrogen bond acceptor and donor properties causes low bioavailability and greatly limits penetration of the blood-brain barrier [16,17]. Among others, these compounds bind to the heme iron in CYP enzymes [18], and when co-administrated with other interacting drugs, can lead to adverse side-effects through drug-drug interactions [19]. Following these discoveries, and the successful cloning of the H_3_ receptor by Lovenberg et al. [20] in 1999, the intensive search for non-imidazole-based compounds was resumed, as these compounds may offer improvements in binding affinity and CNS penetration [21,22]. Despite significant differences in molecular weight and polarity, a general pharmacophore model has been developed for non-imidazole H_3_-antagonists. They are usually characterized by a basic group, often a tertiary amine, connected through an aliphatic spacer to a second pharmacophoric fragment which includes, a lipophilic substituent (often with a phenyl-like structure), depending on the series; some pronounced examples where the phenyl moiety was successfully replaced by different heterocyclic rings, one being a structurally modified thiazole ring are given in the literature [23,24]; (UCBPharma and compound **1**, Figure 1). The lipophilic part is accompanied by another displaying high chemical diversity, such as an H-donor/acceptor, a 2nd basic part or acid group or lipophilic residue [25]. These efforts have resulted in a number of non-imidazole antagonists with high selectivity and specificity. The successful replacement of the imidazole moiety with pyrrolidine, piperidine, piperazine and other basic tertiary amines has been reported in patent applications and chemistry papers [26,27,28,29]. Recently, compound **BF2.649** (Wakix^®^), an H_3_ inverse agonist, carrying the characteristic 3-aminopropan-1-ol functionality in its structure, has undergone clinical studies, and been approved and registered as a drug against narcolepsy [30]. Our previous study described two series of *N*-methyl-2-[2-(4-propylpiperazin-1-yl)-1,3-thiazol-5-yl]- [31] and *N*-methyl-2-[2-(4-propylpiperazin-1-yl)-1,3-thiazol-4-yl]ethan-1-amine derivatives [32] in which the terminal secondary *N*-methylamino function has been substituted with ω-aliphatic and ω-phenylaliphatic moieties (carbon chains of varying lengths from one to five methylene groups) with moderate to pronounced affinity for the histamine H_3_ receptor. It was shown by comparison of the homologous pairs of both series, that the presence of the aforementioned substituents at position 5 in the thiazole ring is favorable for histamine H_3_ receptor antagonist activity, whereas substitution at position 4 typically leads to a strong decrease of activity. The highest affinity for these series was seen for the derivative bearing *N*-methyl-*N*-phenylpropyl moiety [31], (compound **ADS-531**; pA_2_ = 8.27; Figure 1) and slightly lower affinity for the one carrying an *N*-methyl-*N*-benzyl or *N*-methyl-*N*-phenylethyl substituent [31] (**2a**, pA_2_ = 7.76; **2b**, pA_2_ = 7.61). The most active compound **ADS-531**, which did not exhibit any H_1_-antagonistic activity, was chosen as the lead compound for further structural modification.

The aim of the present study was, hence, to optimize the structure of the lead compound **2c**. To this end, a series of 5-substituted-2-thiazol-4-*n*-propylpiperazines (**3**; Figure 1) were synthesized and their pharmacological properties functionally evaluated with an in vitro test system using guinea pig jejunum preparations [33]. In this series, the alkyl chain between position 2 of the thiazole ring and the terminal secondary *N*-methylamino function was elongated from three to four methylene groups. The *N*-methylamino group was substituted by benzyl-, 2-phenylethyl-, and 3-phenylpropyl- substituents, these substituents being found to have the highest potency in previously described series of thiazoles [31]. Furthermore, compounds with the highest potency at the H_3_ receptor were also tested for H_1_ antagonistic effects in vitro, according to standard methods, using guinea pig ileum [34].

The results of SARs, together with previously described data, indicated that compound **ADS-531**, devoid of the antagonistic activity at the H_1_ receptor, was the most active of the group of compounds **2a**–**c**. This compound was evaluated for its affinity for the recombinant rat and human histamine H_3_ receptors (rH_3_R and hH_3_R, respectively), transiently expressed in HEK-293T cells. Additionally, derivative **ADS-531** was subjected to in vivo evaluation of its impact on feeding behavior and brain neurotransmitter systems after repeated peripheral administration to rats. Postmortem analyses of the rat brain tissues were also carried out to determine the activities of MAO-A, MAO-B, and HNMT.

## 2. Results and Discussion

### 2.1. Chemistry

**Scheme 1:**
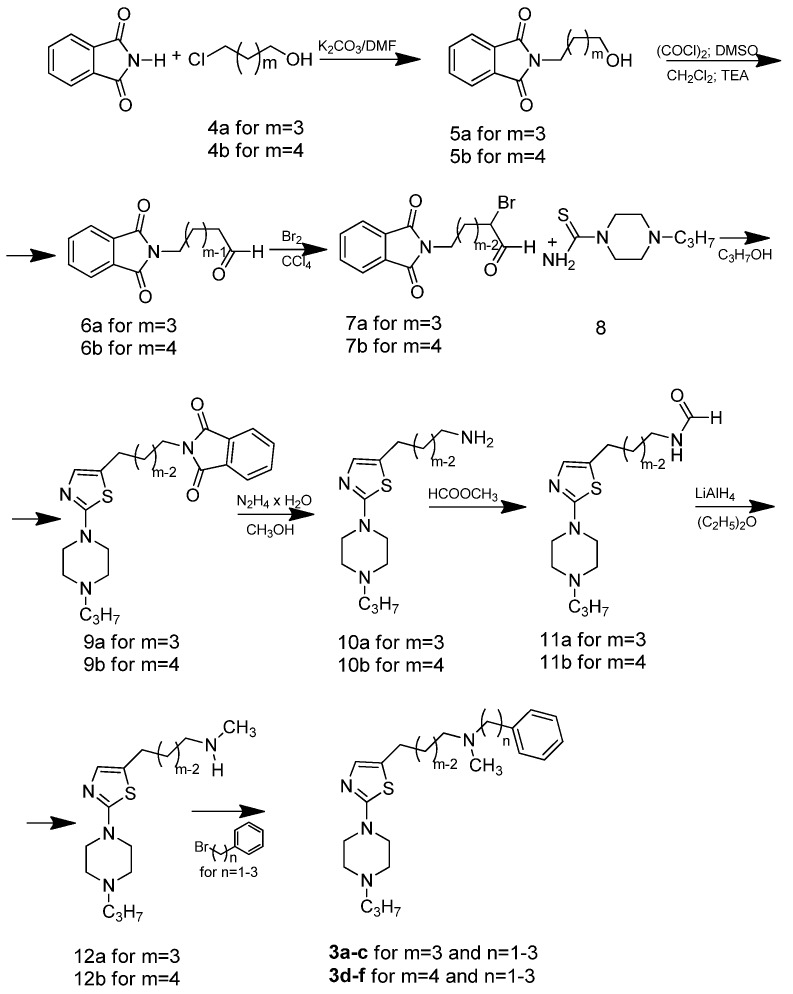
Synthetic routes to compounds **3a**–**f**.

The general synthetic procedure used in this study is illustrated in Scheme 1. The central building blocks of the title compounds (compounds **3a**–**c** and **3d**–**f**) were 3-[2-(4-propylpiperazin-1-yl)-1,3-thiazol-5-yl]propan-1-amine (**10a**) and 4-[2-(4-propylpiperazin-1-yl)-1,3-thiazol-5-yl]butan-1-amine (**10b**), respectively (Scheme 1). The phthalimidobutanols **5a** and **5b** were prepared from ω-chloroalkanols **4a** and **4b** and phthalimide to yield 70% and 62% of the desired compounds, respectively.

The ω-phthalimidoalkanals **6a** and **6b** were obtained from ω-phthalimidoalkanols **5a** and **5b** by reaction with oxalyl chloride/dimethyl sulfoxide, followed by proton abstraction with triethylamine and hydrolysis to the corresponding aldehydes. Bromination of the aldehydes was performed in carbon tetrachloride and after identification with NMR, the crude 2-bromo-ω-phtalimidoalkanals **7a** and **7b** were used in the cyclization reaction (Scheme 1). Ring closure of the crude 2-bromo-ω-phtalimidoalkanals **7a** and **7b** with 1-(4-*n*-propyl)piperazine thioamide (**8**) was performed in anhydrous DMF under an argon atmosphere giving high yields of the desired 5-(ω-phthalimidoalkyl)thiazoles **9a**, **9b**. Subsequent hydrazinolysis, basification with sodium hydroxide and extraction with chloroform led to the production of pure amines **10a**, **10b**. Derivatives **12a** and **12b** were prepared from compound **10a**, **10b** by two-step synthesis including formylation with methyl formate to compounds **11a**, **11b** and finally reduction with LiAlH_4_ in dry ethyl ether. Propan-1-amines **3a**–**c** and butan-1-amines **3d**–**f** were synthesized from compounds **12a**, **12b** by alkylation with the corresponding primary phenyloalkyl halides in the presence of K_2_CO_3_ in DMF followed by purification by column chromatography. All free bases were treated with methanolic HBr and the hydrobromides were precipitated with dry diethyl ether. The 1-(4-*n*-propyl)piperazine thioamide (**8**) was directly obtained by the reaction of the 1-*n*-propylpiperazine dihydrobromidewith potassium thiocyanate in aqueous solution [31]. ^1^H- and ^13^C-NMR spectra for all newly synthesized compounds are shown in the Supplementary Material.

6-Chlorohexanol, 7-chloroheptanol, phthalimide, oxalyl chloride, dimethyl sulfoxide (DMSO), 1-*n*-propylpiperazinedihydrobromide, triethylamine, methyl formate, lithium aluminum hydride(LiAlH_4_), benzyl bromide, 1-bromo-2-phenylethane and 1-bromo-3-phenylpropane and all solvents were purchased from Sigma-Aldrich (Saint Louis, MO, USA) or Alfa Aesar (Haverhill, MA, USA) and used without any purification.

### 2.2. Pharmacology

#### 2.2.1. In Vitro Pharmacological Studies

##### H_3_ Antagonistic Activity for Compounds **2a**–**c** and **3a**–**f**

The compounds were tested in vitro as H_3_ receptor antagonists against H_3_ agonist-induced inhibition of the electrically evoked contraction of the guinea-pig jejunum [33]. The potency of the newly synthesized compounds **3a**–**f** are reported in Table 1, as well as the previously described data for compounds **2a**,**c** [31] and **2b** [32]. Derivatives **3a**–**f** show moderate to pronounced antagonist activity at H_3_-receptor. Propan-1-amines **3a**–**c** (Table 1) and butan-1-amines **3d**–**f** (Table 1) were synthesized to optimize the structure of the lead compound **ADS-531** and the complementary **2a**–**c** derivatives series [31,32]. In this series, the alkyl chain between position 2 of the thiazole ring and the terminal secondary *N*-methylamino function was elongated from three to four methylene groups. The *N*-methylamino functionality was substituted by benzyl-, 2-phenylethyl-, and 3-phenylpropyl- substituents, with these showing the highest potency in previously described series **2a**–**c**. A comparison of homologous triplets, carrying benzyl substituents (compounds **2a**, **3a**, **3d**), found derivative **3a** (pA_2_ = 8.38) to have a higher potency than its analogs **2a**, **3d** (pA_2_ = 7.76 and 7.46, respectively). In the case of derivatives bearing a 2-phenylethyl substituent (compounds **2b**, **3b**, and **3e**) the potency increases slightly with increasing alkyl chain length (pA_2_ = 7.61, 7.81 and 7.95, respectively). The highest potency, for the series of derivatives carrying of 3-phenylpropyl- substituent (compounds **2c**, **3c**, and **3f**), is seen for **2c** (pA_2_ = 8.27), but an increase in the alkyl chain length to two methylene groups resulted in a decrease of antagonist activity for compound **3c** (pA_2_ = 7.46), while the activity increased again when the chain was further lengthened to three methylene groups (**3f**; pA_2_ = 7.91).

Differences are observed within the **2a**–**c** and **3a**–**c** series. In the series of derivatives containing an ethyl linker between position 2 of the thiazole ring and the terminal secondary *N*-methylamino function (compounds **2a**–**c**), the compound bearing a 3-phenylpropyl-residue (**2c**; pA_2_ = 8.27) shows the highest potency at the H_3_-receptor. Shortening the alkyl chain to two methylenes (compound **2b**) or one methylene group (compound **2a**) leads to a compound with a lower potency (pA_2_ = 7.61 and pA_2_ = 7.76, respectively). These results are in contrast to the results obtained for a series containing a propyl linker (compounds **3a**–**c**). The compound carrying a benzyl substituent (**3a**; pA_2_ = 8.38) shows the highest potency at the H_3_ receptor, while the derivative with a 3-phenylpropyl moiety (**3c**; pA_2_ = 7.46) shows the lowest antagonist activity. Compounds **3d**–**f**, containing a butyl linker, show moderate potency at H_3_-receptor, independent of the alkyl chain length in the ω-phenylalkyl substituent (pA_2_ ranging from 7.91 to 7.97).

To summarize, the obtained results indicated that elongation of the alkyl chain from two to three methylene groups between position 2 of the thiazole ring and the terminal secondary *N*-methylamino function resulted in compound **3a** (bearing a benzyl substituent, and propyl linker). This compound demonstrated slightly higher potency than the parent compound **2c** (bearing a 3-phenylpropyl moiety, and an ethyl linker) but in contrast to **3a**, **2c** did not possess any activity at H_1_.

##### H_1_ Antagonistic Activity for Compounds **3a**, and **3d**

The final compounds showing the highest potency for the H_3_ receptors were also tested for H_1_ antagonistic effects in vitro, following standard methods, using the guinea pig ileum [34]. Compounds **3a**, and **3d** show weak, but competitive H_1_-antagonistic activity with pA_2_ = 5.5, and pA_2_ = 6.25 (Table 1), respectively (for pyrilamine pA_2_ = 8.66).

#### 2.2.2. Histamine H_3_ Receptor Affinity

Additionally, the affinity of the most active compound **ADS-531** was evaluated by measuring the displacement curve of [^3^H]-*N*^α^-methylhistamine at the rat (rH_3_R) and human histamine H_3_ receptor (hH_3_R) in HEK-293Tcell membranes as described by Bongers [35].

##### Saturation of Rat and Human H_3_ Receptors

To determine the total and non-specific binding, membranes expressing rH_3_R or hH_3_R were incubated with different concentrations of [^3^H]-*N*^α^-MH (0–20 nM) in the absence or presence of unlabeled thioperamide (10 μM) for two hours at 25 °C. The reaction was terminated by rapid filtration on GF/C 96 well plates and the levels of the bound radioligand were measured by scintillometry. Specific binding was defined as the difference between the total and non-specific binding conditions. A representative graph of the saturation of rat and human H_3_R can be found in the Supplementary Material.

Analysis of the [^3^H]-*N*^α^-MH saturation binding yielded at rH_3_R a *K*_D_ value of 2.72 ± 0.34 nM and a *B*_max_ value of 2715 ± 445 fmol/mg protein and at hH_3_R a *K*_D_ value of 0.9 ± 0.08 nM and a *B*_max_ value of 632 ± 52 fmol/mg protein.

##### Competitive Binding of H_3_ Receptor Ligands

The affinity of **ADS-531**, histamine and thioperamide—the reference compound—were determined by measuring the displacement curves of [^3^H]-*N*^α^-methylhistamine binding to the rat and human histamine H_3_ receptor expressed in HEK-293T membranes. Derivative **ADS-531** possesses a slightly lower nanomolar affinity for the rat H_3_R (pK_i_ 7.5 ± 0.1) than thioperamide (pK_i_ 7.9 ± 0.1), and slightly higher than histamine (pK_i_ = 7.3 ± 0.1). A significantly higher affinity is observed for **ADS-531** at the human H_3_R (pK_i_ 8.5 ± 0.1) than of thioperamide (pK_i_ 7.2 ± 0.1) and pK_i_ of histamine (7.7 ± 0.1). Representative graphs of competition binding of H_3_R ligands on the rat and human H_3_ receptors are shown in the Supplementary Material (Section 2.2.2; Figures S2 and S3, respectively).

#### 2.2.3. Verification of In Vivo Activity of Compound **ADS-531**

The brain histaminergic system participates in the regulation of feeding behavior, and of the four histamine receptors, H_1_ and H_3_ play an important role. Their activation is a critical part of the regulatory mechanism behind the diurnal rhythm of food consumption, as well as energy intake and expenditure [36,37,38,39]. Studies have shown that the central administration of histamine and likewise H_1_ receptor agonists, lowered food intake. Also, the strategies leading to enhanced synaptic histamine availability—i.e., the blockade of the H_3_ receptor or inhibition of histamine catabolism—caused hypophagia while the administration of H_1_ antagonists resulted in hyperphagia [37]. An in vivo evaluation was therefore performed on the impact of compound **ADS-531** on brain neurotransmitter systems. Given that the compound enters the CNS and blocks H_3_R, its peripheral administration should result in neuronal histamine release. The released histamine, in turn, acting via H_1_R, would induce loss of appetite, resulting in a decrease of food intake. To ensure conclusive results, a five-day course of treatment was chosen with daily monitoring of consumption at 9 a.m. Any influence of subchronic administration of **ADS-531** on cerebral amine neurotransmitters concentrations and/or the activities of catabolic enzymes, monoamine oxidases A and B and histamine *N*-methyltransferase would be disclosed by post-mortem analyses of the brain tissues of the treated rats. In our in vivo studies, Lewis rats were used as subjects, and ciproxifan was used as a reference instead of thioperamide, because the latter demonstrated lower bioavailability due to restricted brain penetration [40].

**Figure 2:**
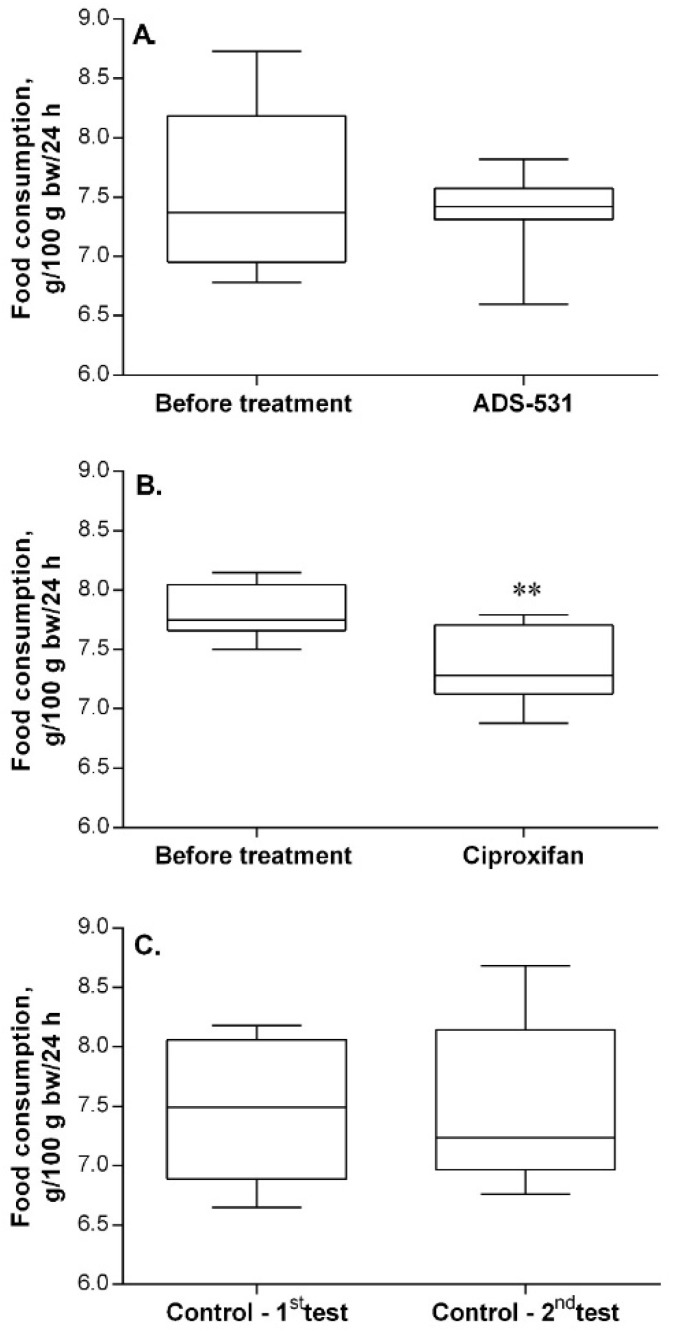
The effect of **ADS-531** (s.c. 3 mg/kg/daily for five days), the newly synthesized histamine H_3_ receptor antagonist (chart **A**), and of the reference ciproxifan (s.c. 3 mg/kg/daily for five days; chart **B**) on food consumption. Consumption by untreated rats (chart **C**). Median (the line in the middle of the box) and the range of values (whiskers) are given for eight rats. Paired *t*-test, ** *p* < 0.01 vs. before treatment.

As can be seen in Figure 2, five-day treatment with **ADS-531** did not influence food intake by the rats, whereas treatment with ciproxifan caused a significant decrease in consumption. It is important to note that the treatment was preceded by an adaptive period to experimental conditions.

**Figure 3:**
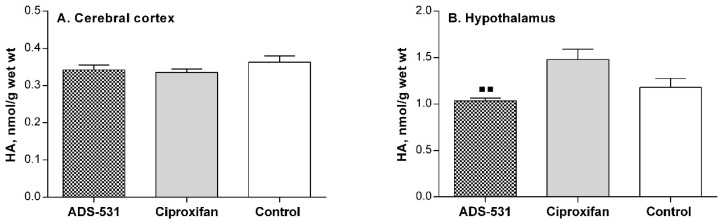
Cerebral histamine concentration in rats subchronically treated with newly synthesized **ADS-531** (s.c. 3 mg/kg/daily for 5 days) histamine H_3_ receptor antagonist or the reference ciproxifan (s.c. 3 mg/kg/daily for five days). One-way ANOVA and Tukey’s multiple comparisons tests: ^■■^ vs. ciproxifan, *p* < 0.01.

No significant changes were observed in CNS histamine concentration (Figure 3), nor in the enzyme activities related to histamine catabolism in the brain tissue of the sacrificed animals (Table 2). In the hypothalamus (Figure 3B), where the histamine cell bodies are located, the amine concentration in the ciproxifan-treated rats tended to be higher than in the untreated controls and was significantly higher than in **ADS-531** injected rats. This could suggest some enhancement of histamine synthesis by ciproxifan, following the presumed enhanced release of histamine to the synapses with its anorectic effects; this observation agrees closely with the observed intravital decrement of food intake (Figure 2B). Histamine is metabolized in the mammalian brain exclusively by the *N*-methylation pathway, involving histamine *N*-methyltransferase at the first step, followed by monoamine oxidase B, which catalyzes the oxidative deamination of *N*-telemethylhistamine. Neither of the two drugs used affected this pathway (Table 2).

**Figure 4:**
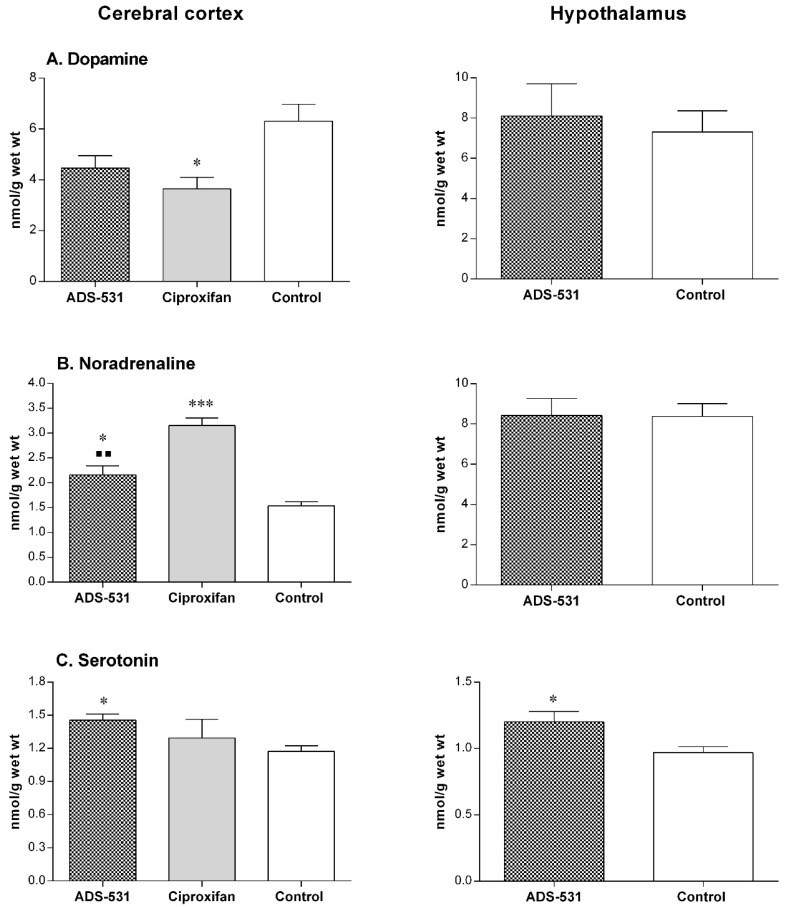
The cerebral concentration of biogenic amines’ in rats subchronically treated with reference Ciproxifan (s.c. 3 mg/kg/daily for five days) and **ADS-531** (s.c. 3 mg/kg/daily for five days), the newly synthesized histamine H_3_ receptor antagonist. DA—dopamine, NA—noradrenaline, 5-HT—serotonin. The values are means ± SEM for five to eight rats. One-way ANOVA and Tukey’s multiple comparisons test (cerebral cortex) or Unpaired *t* test (hypothalamus): * vs. Control, ^■^ vs. Ciproxifan; a single symbols means *p* < 0.05, two symbols: *p* < 0.01, three symbols: *p* < 0.001.

One way ANOVA and Tukey’s multiple comparisons test showed no statistically significant differences. The apparent lack of the effects of the tested compound on the histaminergic system was by no means caused by the lack of its ability to cross the blood-brain barrier. The data presented in Figure 4 clearly indicates that **ADS-531** caused alterations in the concentrations of dopamine, noradrenaline, and serotonin.

An increase of 5HT and NA level throughout the brain with the exception of the hypothalamus indicates decreased serotonergic and noradrenergic activity and a concomitant increase in DA system activity. These findings may suggest that **ADS-531** is likely to show some agonistic activity to serotonin autoreceptors, thereby modifying dopamine and noradrenaline release [41].

## 3. Conclusions

**ADS-531** was found to exhibit the highest in vitro affinity toward the H_3_ guinea pig jejunal receptors with pA_2_ = 8.27. In competition radioligand binding studies at the rat histamine H_3_ receptor, compound **ADS-531** (pK_i_ 7.5 ± 0.1) showed slightly lower nanomolar affinity than the reference compound—thioperamide (pK_i_ = 7.9), and slightly higher than histamine (pK_i_ = 7.3 ± 0.1). Significantly higher affinity was observed for **ADS-531** at the human H_3_R (pK_i_ = 8.5 ± 0.1) than thioperamide (pK_i_ = 7.2 ± 0.1) and pK_i_ of histamine (7.7 ± 0.1). **ADS-531** given parenterally for five days did not influence the food intake in rats. No significant changes were observed in histamine concentration, nor in the enzyme activities related to histamine metabolism examined in the brain. The apparent lack of the effects of the tested compound on the histaminergic system was by no means caused by the lack of its ability to cross the blood-brain barrier. The presented data leaves no doubt that **ADS-531** caused alterations in the concentrations of dopamine, noradrenaline, and serotonin. The high potency and affinity for H_3_ receptors and in vivo activity suggest that further study on **ADS-531** is merited.

## 4. Experimental Section

### 4.1. General Information 

All melting points (m.p.) were measured in open capillaries on an electrothermal apparatus and are uncorrected. Infrared spectra (IR) were measured on a FT-IR vegus spectrophotometer (Thermo Nicolet, city, state abbrev if USA, country). For all compounds, ^1^H-NMR spectra were recorded on a Mercury VX 300 MHz spectrometer (Varian, city, state abbrev if USA, country). Chemical shifts are expressed in ppm downfield from internal TMS as a reference. ^1^H-NMR data are reported in order: multiplicity (br, broad; s, singlet; d, doublet; t, triplet; m, multiplet; * exchangeable by D_2_O) number of protons, and approximate coupling constant in Hertz. ^13^C-NMR spectra were recorded on an Avance III 600 MHz spectrometer (Bruker, city, state abbrev if USA, country). Elemental analysis (C, H, N) for all compounds was measured on Series II CHNS/O Analyzer 2400 (Perkin Elmer, city, state abbrev if USA, country) and were within ±0.4% of theoretical values. TLC was performed on silica gel 60 F_254_ plates (Merck, city, state abbrev if USA, country). Flash column chromatography was carried out using silica gel 60Å 50 μm (J. T. Baker B. V., Phillipsburg, NJ, USA), employing the same eluent as was indicated by TLC. All obtained final free bases were treated with methanolic HBr, the hydrobromide was precipitated with dry diethyl ether and crystallized twice from ethanol.

### 4.2. Chemistry

#### 4.2.1. General Procedure for the Preparation of Compounds **5a**,**b**

A solution of phthalimide (14.7 g, 0.1 mol) and 5-chloropentanol (11.25 g, 0.1 mol) or 6-chloro-hexanol (12.45 g, 0.1 mol) in anhydrous DMF (100 mL) was heated at 150 °C with vigorous stirring in the presence of finely-powdered anhydrous K_2_CO_3_ (13.8 g, 0.1 mol) for 12 h. After cooling, the inorganic materials were filtered off and the solvent was evaporated in vacuo (2 mmHg, 100 °C). The residue was dissolved in 150 mL of ethyl acetate, and after standing overnight at 5 °C, the solution was filtered and concentrated in vacuo. The residue was taken up in 80 mL of CHCl_3_. After extraction with 60 mL of 5% aqueous solution of NaHCO_3_ and 3 × 60 mL of H_2_O, the solvent was dried over Na_2_SO_4_ and removed in vacuo, giving:

*2-(5-Hydroxypentyl)-1H-isoindole-1,3(2H)-dione* (**5a**). A sticky oil (on standing for a prolonged period the viscous oil crystallized giving a solid), yield 70% (16.32 g); m.p. 41–43 °C; R_f_ = 0.39 (CHCl_3_/CH_3_OH 20:1); ^1^H-NMR (300 MHz, CDCl_3_): δ = 1.40–1.43 (m, 4H, CH_2_C), 1.53–1.58 (m, CH_2_CH_2_OH), 1.66–1.71 (m, 3H, CH_2_CH_2_N, OH), 3.61–3.63 (t, *J* = 6.6 Hz, 2H, CH_2_N), 3.66–3.68 (t, *J* = 7.2 Hz, 2H, CH_2_OH), 7.68–7.71 (m, 2H, H_arom_); 7.81–7.84 (m, 2H, H) ppm.

*2-(6-Hydroxyhexyl)-1H-isoindole-1,3(2H)-dione* (**5b**). A sticky oil, yield 62% (15.33 g); R_f_ = 0.40 (CHCl_3_/CH_3_OH 20:1); ^1^H-NMR (300 MHz, CDCl_3_): δ = 1.40–1.43 (m, 4H, CH_2_C), 1.53–1.58 (m, CH_2_CH_2_OH), 1.66–1.71 (m, 3H, CH_2_CH_2_N, OH), 3.61–3.63 (t, *J* = 6.6 Hz, 2H, CH_2_N), 3.66–3.68 (t, *J* = 7.2 Hz, 2H, CH_2_OH), 7.68–7.71 (m, 2H, H), 7.81–7.84 (m, 2H, H) ppm.

#### 4.2.2. General Procedure for the Preparation of Compounds **6a**,**b**

To a well-stirred solution of oxalyl chloride (6.08 mL, 0.07 mol) in dry CH2Cl2 (120 mL), a solution of anhydrous DMSO (5.69 mL, 0.08 mol) in CH_2_C1_2_ (200 mL) was added under an argon atmosphere at −70 °C at such a rate that the temperature was maintained at −70 °C. After the addition was completed, stirring was continued for 15 min, and then, a solution of **5a** (7.7 g, 0.033 mol) or **5b** (8.16 g, 0.033 mol) in dry CH_2_C1_2_ (70 mL) was added while keeping the temperature at −70 °C. The reaction mixture was stirred for another 30 min at −70 °C, and Et_3_N (17.1 mL, 0.122 mol) was added. The mixture was allowed to warm to ambient temperature, and 100 mL of 5% aqueous solution of HCl was added and stirring was continued for 20 min. The organic layer was separated and extracted with H_2_O to the almost neutral reaction, dried over anhydrous Na_2_SO_4_, filtered, and concentrated in vacuo to yield the crude product **6a** and **6b**. The obtained viscous oils were stored under argon and used without further purification for the preparation of **7a** and **7b**:

*5-(1,3-Dioxo-1,3-dihydro-2H-isoindol-2-yl)pentanal* (**6b**). A sticky oil, 82.0% (7.63 g) based on ^1^H-NMR); R_f_ = 0.25 (CHCl_3_/CH_3_OH 100:1); ^1^H-NMR (300 MHz, CDCl_3_): δ = 1.62–1.77 (m, 4H, CCH_2_CH_2_C), 2.50–2.52 (t, *J* = 7.2, *J* = 1.8 Hz, 2H, CH_2_CHO), 3.64–3.72 (t, *J* = 7.2 Hz, CH_2_N), 7.69–7.74 (m, 2H, H ar.), 7.82–7.86 (m, 2H, H), 9.69–9.71 (t, *J* = 1.8 Hz, CHO) ppm.

*6-(1,3-Dioxo-1,3-dihydro-2H-isoindol-2-yl)hexanal* (**6b**). A sticky oil, 79.0% (8.09 g) based on ^1^H-NMR); R_f_ = 0.46 (CHCl_3_/CH_3_OH 100:1); ^1^H-NMR (300 MHz, CDCl_3_): δ ppm: 1.28–1.35 (m, 2H, CH_2_C), 1.58–1.66 (m, 4H, CH_2_CH_2_CHO, CH_2_CH_2_N), 2.34–2.39 (m, 2H, CH_2_CHO), 3.58–3.62 (t, *J* = 7.2 Hz, CH_2_N), 7.62–7.68 (m, 2H, H), 7.24–7.78 (m, 2H, H), 9.67–9.68 (t, *J* = 1.8 Hz, CHO) ppm.

#### 4.2.3. General Procedure for the Preparation of Compounds **7a**,**b**

Small portions of a solution of Br_2_ (4.8 g, 0.03 mol) in acetonitrile (10 mL) were added to a stirred solution of **6a** (6.93 g, 0.03 mol) or **6b** (7.36 g, 0.03 mol) in CCl_4_ (130 mL) under an argon atmosphere. The reaction mixture was stirred for two hours at ambient temperature, and then 5% aqueous solution of NaHCO_3_ (200 mL) and CHCl_3_ (50 mL) were added followed by stirring for 15 min. The organic phase was separated and extracted under an argon atmosphere with H_2_O till neutral reaction. After drying over anhydrous Na_2_SO_4_ and filtration, the solvent was removed in vacuo. The resulting viscous oils were used without further purification for the preparation of **9a** and **9b**.

*2-Bromo-5-(1,3-dioxo-1,3-dihydro-2H-isoindol-2-yl)pentanal* (**7a**). A sticky oil (containing 9.3 g of **7a**), yield 76.0% (based on ^1^H-NMR); R_f_ = 0.44 (CHCl_3_/CH_3_OH 100:1); ^1^H-NMR (300 MHz, CDCl_3_): δ = 2.08–2.13 (m, 2H, CH_2_CH_2_CH_2_), 2.49–2.54 (m, 2H, CH_2_CHBr), 3.74–3.76 (t, *J* = 7.2 Hz, 2H, CH_2_N), 4.33–4.36 (m, 1H, CHCHO), 7.72–7.74 (m, 2H), 7.84–7.87 (m, 2H), 9.44 (d, *J* = 2.4 Hz, 1H, CHO) ppm.

*2-Bromo-6-(1,3-dioxo-1,3-dihydro-2H-isoindol-2-yl)hexanal* (**7b**). A sticky oil (containing 9.7 g of **7b**) yield 74.7% (based on ^1^H-NMR); R_f_ = 0.46 (CHCl_3_/CH_3_OH 100:1); ^1^H-NMR (300 MHz, CDCl_3_): δ = 1.72–1.80 (m, 4H, CH_2_C), 1.96–2.11 (m, 2H, CH_2_CHBrCHO), 3.68–3.73 (m, 2H, CH_2_N), 4.20–4.26 (m, 1H, CHBr), 7.82–7.85 (m, 2H, H_arom_.), 7.85–7.87 (m, 2H, H_arom_.), 9.43–9.44 (d, *J* = 2.7 Hz, 1H, CHO) ppm.

#### 4.2.4. General Procedure for the Preparation of Compounds **9a**,**b**

A solution of 4-propylpiperazine-1-carbothioamide (**8**, 4.12 g, 0.022 mol) in anhydrous DMF (250 mL) was added to a stirred solution of **7a** (7.06 g, 0.022 mol) or **7b** (7.2 g, 0.022 mol) in anhydrous DMF (250.0 mL) under an argon atmosphere. The reaction mixture was heated at 95 °C for nine hours. After cooling, the solvent was removed in vacuo. The residue was dissolved in *n*-propanol and cooled to 5 °C. The precipitate was filtered off and washed with ether. The hydrobromide product was obtained as a brown solid. The free base was obtained as follows: the hydrobromide was mixed with saturated aqueous potassium carbonate solution over night at room temperature. The solid was filtered, washed with water, ether and air dried to leave a light brown solid. The crude product was purified by silica gel flash-column chromatography, giving:

*2-[3-(4-n-Propylpiperazin-1-yl)propyl]-1H-isoindole-1,3(2H)-dione* (**9a**). A white solid, yield 34% (2.98 g); R_f_ = 0.51 (CHCl_3_/CH_3_OH 15:1); m.p.: 80–81 °C; (m.p._dihydrobromide_: 258–260 °C), ^1^H-NMR (300 MHz, CDCl_3_): δ = 0.89–0.94 (t, *J* = 7.2 Hz, 3H, CH_3_); 1.46–1.56 (sx, *J* = 7.2 Hz, 2H, CH_3_CH_2_), 1.97–2.02 (p, *J* = 7.2 Hz, 2H, CH_2_CH_2_N), 2.32–2.35 (m, 2H, CH_2_N), 2.49–2.58 (m, 4H, CH_2piperazine_), 2.72–2.74 (t, *J* = 7.2 Hz, 2H, CH_2thiazole_), 3.41–3.43 (m, 4H, CH_2piperazine_), 3.75–3.76 (t, *J* = 7.2 Hz, CHN(CO)_2_), 6.87 (s, 1H, H_thiazole_), 7.70–7.72 (m, 2H, H_arom_), 7.82–7.84 (m, 2H, H_arom._) ppm.

*2-[4-(4-n-Propylpiperazin-1-yl)butyl]-1H-isoindole-1,3(2H)-dione* (**9b**). A white solid, yield 38% (3.44 g); R_f_ = 0.23 (CHCl_3_/CH_3_OH 20:1); m.p.: 102–104 °C; (m.p._dihydrobromide_: 257–259 °C); ^1^H-NMR (300 MHz, CDCl_3_): δ = 0.89–0.94 (t, *J* = 7.2 Hz, 3H, CH_3_), 1.49–1.74 (m, 6H, CH_2_C), 2.30–2.35 (m, 2H, CH_2_N), 2.52–2.54 (m, 4H, H_piperazine_), 2.66–2.69 (m, 2H, CH_2thiazole_), 3.42–3.44 (m, 4H, H_piperazine_), 3.67–3.69 (t, *J* = 7.2 Hz, CH_2_NCO), 6.80–6.81 (t, *J* = 1.2 Hz, CH_thiazole_), 7.68–7.70 (m, 2H, H_arom._); 7.79–7.82 (m, 2H, H_arom_) ppm.

#### 4.2.5. General Procedure for the Preparation of Compounds **10a**,**b**

A solution of **9a** (3.19 g, 0.008 mol) or **9b** (3.30 g, 0.008 mol) and of N_2_H_4·_H_2_O (0.8 g, 0.016 mol) in MeOH (50 mL) was refluxed for nine hours. The solvent was evaporated and the remaining material was dissolved in 30 mL of methylene chloride. After cooling, the crystallized phthalhydrazide was filtered off. Concentration in vacuo provided a white sticky semi-solid, which was purified by column chromatography on silica gel, giving:

*3-[2-(4-Propylpiperazin-1-yl)-1,3-thiazol-5-yl]propan-1-amine* (**10a**). A sticky oil, yield 60% (1.29 g); R_f_ = 0.33 (CHCl_3_/MeOH/NH_3_aq 60:10:1); m.p._treehydrobromide_: 240–242 °C; ^1^H-NMR (300 MHz, CDCl_3_) δ = 0.91–0.93 (t, *J* = 7.2 Hz, 3H, CH_3_), 1.39 (s_br_, 2H, NH_2_), 1.49–1.56 (sx, *J* = 7.2 Hz, 2H, CHCH_2_), 1.71–1.76 (p, *J* = 7.2 Hz, 2H, CCH_2_C), 2.33–2.36 (m, 2H, CH_2_N), 2.52–2.54 (m, 4H, CH_2piperazine_), 2.70–2.72 (t, *J* = 7.2 Hz, 2H, CH_2thiazole_), 2.74–2.76 (t, *J* = 7.2 Hz, 2H, CH_2_NH_2_), 3.43–3.45 (m, 4H, CH_2piperazine_), 6.84 (s, 1H_thiazole_) ppm.

*4-[2-(4-Propylpiperazin-1-yl)-1,3-thiazol-5-yl]butan-1-amine* (**10b**). A sticky oil, yield 62% (1.40 g): R_f_ = 0.18 (CHCl_3_/MeOH/NH_3_aq 60:10:1); m.p._treehydrobromide_: 222–224 °C; ^1^H-NMR (300 MHz, CDCl_3_) δ = 0.87–0.92 (t, *J* = 7.5 Hz, 3H, CH_3_), 1.45–1.63 (m, 6H, CH_2_C), 2.25 (s_br_, 2H, NH_2_), 2.29–2.35 (m, 2H, CH_2_N_-propyl_), 2.50–2.53 (m, 4H, H_piperazine_), 2.62–2.72 (m, 4H, CH_2thiazole_, CH_2_N), 3.40–3.43 (m, 4H, CH_2piperazine_), 6.81 (s, 1H, CH_thiazole_) ppm.

#### 4.2.6. General Procedure for the Preparation of Compounds **11a**,**b**

A solution of **10a** (1.85 g, 0.0069 mol) or **10b** (1.95 g, 0.0069 mol) and methyl formate (16.33 g, 16.0 mL, 0.27 mol) in MeOH (6 mL), was heated under an argon atmosphere at 32 °C for 48 h. The solvent was evaporated to yield the crude product **11a** and **11b**. The obtained viscous oils were stored under argon and used without further purification for the preparation of **7a** and **7b**.

*N-{3-[2-(4-Propylpiperazin-1-yl)-1,3-thiazol-5-yl]propyl}formamide* (**11a**). A sticky oil (on standing for a prolonged period the viscous oil crystallized giving the solid, m.p. 57–59 °C), yield 91.0% (1.87 g; based on ^1^H-NMR); R_f_ = 0.5 (CHCl_3_/CH_3_OH 9:1); ^1^H-NMR (300 MHz, CDCl_3_) δ = 0.91–0.93 (t, *J* = 7.2 Hz, 3H, CH_3_), 1.50–1.56 (sx, *J* = 7.2 Hz, 2H, CH_3_CH_2_), 1.80–1.85 (p, *J* = 7.2 Hz, 2H, CCH_2_C), 2.32–2.36 (m, 2H, CH_2_N), 2.53–2.55 (m, 4H, CH_2piperazine_), 2.69–2.72 (t, *J* = 7.2 Hz, 2H, CH_2thiazole_), 3.32–3.35 (q, *J* = 6.6 Hz, 2H, CH_2_NHCHO), 3.43–3.46 (m, 4H, CH_2piperazine_), 6.06 (br, 1H, NH), 6.84 (s, 1H, H_thiazole_), 8.16 (s, 1H, CHO) ppm.

*N-{4-[2-(4-Propylpiperazin-1-yl)-1,3-thiazol-5-yl]butyl}formamide*(**11b**). A sticky oil (on standing for a prolonged period the viscous oil crystallized giving the solid, m.p. 58–60 °C), yield 83.0% (1.77 g, based on ^1^H NMR); R_f_ = 0.58 (CHCl_3_/CH_3_OH/NH_3aq_ 60:10:1); ^1^H-NMR (300 MHz, CDCl_3_): δ = 0.91–0.93 (t, *J* = 7.4 Hz, 3H, CH_3_), 1.50–1.64 (m, 6H, CH_2_-C), 2.34–2.36 (m, 2H, CH_2_N_piperazine_), 2.53–2.57 (m, 4H, CH_2piperazine_), 2.67–2.69 (t, *J* = 6.7 Hz, CH_2thiazole_), 3.30–3.33 (t, *J* = 7.6 Hz, 2H, CH_2_NH); 3.44–3.45 (m, 4H, CH_2piperazine_); 5.61 (s_br_, 1H, NH); 6.83 (s, 1H, H_thiazole_); 8.16 (s, 1H, CHO) ppm.

*N-{4-[2-(4-Propylpiperazin-1-yl)-1,3-thiazol-5-yl]butyl}formamide* (**11b**). A sticky oil (on standing for a prolonged period the viscous oil crystallized giving the solid, m.p. 58–60 °C), yield 83.0% (1.77 g, based on ^1^H NMR); R_f_ = 0.58 (CHCl_3_/CH_3_OH/NH_3aq_ 60:10:1); ^1^H-NMR (300 MHz, CDCl_3_): δ = 0.91–0.93 (t, *J* = 7.4 Hz, 3H, CH_3_), 1.50–1.64 (m, 6H, CH_2_-C), 2.34–2.36 (m, 2H, CH_2_N_piperazine_), 2.53–2.57 (m, 4H, CH_2piperazine_), 2.67–2.69 (t, *J* = 6.7 Hz, CH_2thiazole_), 3.30–3.33 (t, *J* = 7.6 Hz, 2H, CH_2_NH); 3.44–3.45 (m, 4H, CH_2piperazine_); 5.61 (s_br_, 1H, NH); 6.83 (s, 1H, H_thiazole_); 8.16 (s, 1H, CHO) ppm.

#### 4.2.7. General Procedure for the Preparation of Compounds **12a**,**b**

To a vigorous stirred suspension of **11a** (0.98 g, 0.0033 mol) or **11b** (1.02 g, 0.0033 mol) in anhydrous ethyl ether (200 mL), LiAlH_4_ (0.35 g, 0.009 mol) was added. The mixture was stirred at room temperature for two hours, and quenched by dropwise addition of water (0.7 mL), 5% of NaOH solution (0.6 mL), and water (0.2 mL). The suspension was stirred for 30 min, and filtered. The filter cake was washed with ether (2 × 50 mL). The combined organic extracts were washed with water (3 × 50 mL), dried (Na_2_SO_4_), and filtered. The solvent was evaporated and remaining material was purified by column chromatography on silica gel, giving:

*N-Methyl-3-[2-(4-propylpiperazin-1-yl)-1,3-thiazol-5-yl]propan-1-amine* (**12a**). A white solid, yield 85% (1.15 g); (m.p. 38–40 °C; R_f_ = 0.24 (CHCl_3_/CH_3_OH/NH_3aq_ 60:10:1); ^1^H-NMR (300 MHz, CDCl_3_): δ = 0.91–0.93 (t, *J* = 7.2 Hz, 3H, CH_3_). 1.23 (s_br_, 1H, NH). 1.50–1.55 (sx, *J* = 7.8 Hz, 2H, CH_2_CH_3_), 1.75–1.85 (p, *J* = 7.2 Hz, 2H, CH_2_CH_2_CH_2_), 2.33–2.35 (m, 2H, CH_2_N), 2.42 (s, 3H, CH3N), 2.52–2.55 (m, 4H, CH_2piperazine_), 2.61–2.63 (t, *J* = 7.2 Hz, 2H, CH_2_NHCH_3_), 2.69–2.72 (t, *J* = 7.2 Hz, 2H, CH_2thiazole_), 3.43–3.46 (m, 4H, CH_2piperazine_), 6.84 (s, 1H, H_thiazole_) ppm; IR (nujol): 3301, 2934, 2843, 2789, 1670, 1519, 1451, 1376, 1278, 1232, 1145, 1037, 988, 912, 842, 802, 751 cm^−1^.

*N-Methyl-4-[2-(4-n-propylpiperazin-1-yl)-1,3-thiazol-5-yl]butan-1-amine* (**12b**). A sticky oil, yield 58.3% (0.57 g); R_f_ = 0.16 (CHCl_3_/CH_3_OH/NH_3aq_ 60:10:1); ^1^H-NMR (300 MHz, CDCl_3_): δ = 0.91–0.93 (t, *J* = 7.4 Hz, 3H, CH_3_), 1.50–1.65 (m, 6H, CH_2_C), 2.33–2.35 (m, 4H, CH_2_N_piperazine_), 2.42 (s, 3H, NCH_3_), 2.52–2.54 (m, 4H, CH_2piperazine_), 2.57–2.59 (t, *J* = 7.0 Hz, 2H, CH_2_NCH_3_); 2.65–2.68 (t, *J* = 7.4 Hz, 2H, CH_2thiazole_); 3.43–3.47 (m, 4H, CH_2piperazine_); 6.83 (s, 1H, H_thiazole_) ppm; IR (nujol): 3298, 3045, 2933, 2874, 2845, 2812, 1520, 1453, 1376, 1341, 1277, 1232, 1168, 1145, 1100, 1037, 988, 890, 843, 802, 752 cm^−1^.

#### 4.2.8. General Procedure for the Preparation of Compounds **3a**–**f**

The corresponding phenylalkyl bromide (0.001 mol) was added to a solution of **12a** (0.283 g, 0.001 mol) or **12b** (0.296 g, 0.001 mol) in the presence of potassium carbonate (0.002 mol) in acetonitrile (20.0 mL). The reaction mixture was stirred for ninety-six hours at room temperature. The solvent was evaporated and water (25 mL) was added to the residue the mixture was extracted with dichloromethane (3 × 25 mL). The water layer was discarded and the solvent was dried over Na_2_SO_4_ and filtered. The solvent was evaporated and the crude product was purified by column chromatography on silica gel, giving:

*N-Benzyl-N-methyl-3-[2-(4-n-piperazin-1-yl)-1,3-thiazol-5-yl]propan-1-amine* (**3a**). A sticky oil, yield 74.0% (0.28 g); R_f_ = 0.16 (CHCl_3_/CH_3_OH 6:1); m.p._trihydrobromide_ = 254–255 °C; ^1^H-NMR (300 MHz, CDCl_3_): δ = 0.91–0.93 (t, *J* = 7.2 Hz, 3H, CH_3_), 1.50–1.56 (sx, *J* = 7.8 Hz, 2H, CH_2_CH_3_), 1.77–1.81 (p, *J* = 7.6 Hz, 2H, CH_2_CH_2_CH_2_), 2.17 (s, 3H, CH_3_), 2.32–2.35 (m, 2H, CH_2_N), 2.41–2.43 (t, *J* = 7.2 Hz, 2H, CH_2_NCH_3_), 2.52–2.54 (m, 4H, CH_2piperazine_.), 2.68–2.71 (t, *J* = 7.2 Hz, 2H, CH_2thiazole_), 3.43–3.47 (m, 4H, CH_2piperazine_), 3.51 (s, 2H, CH_2_Ph), 6.82 (s, 1H, H_thiazole_), 7.21–7.24 (m, 1H), 7.28–7.31 (m, 4H) ppm; ^13^C-NMR (75 MHz, CDCl_3_): δ = 11.83, 19.94, 24.68, 29.05, 42.04, 48.38, 52.37, 56.35, 60.53, 62.34, 126.82, 127.49, 128.13, 128.91, 135.41, 139.20, 170.59 ppm. IR (nujol): 3027, 2938, 2874, 2786, 1519, 1453, 1376, 1280, 1231, 1168, 1145, 1004, 911, 845, 737, 699 cm^−1^. Anal. calcd for C_21_H_35_ Br_3_N_4_S (M = 615.34): C 40.99, H 5.73, N9.11, found: C 40.81, H 5.76, N 8.90.

*N-Methyl-N-(2-phenylethyl)-3-[2-(4-n-propylpiperazin-1-yl)-1,3-thiazol-5-yl]propan-1-amine* (**3b**). A sticky oil; yield 82.0% (0.32 g); R_f_ = 0.45 (CHCl_3_/CH_3_OH 6:1); m.p._trihydrobromide_ = 255–256 °C; ^1^H-NMR (300 MHz, CDCl_3_): δ = 0.90–0.93 (t, *J* = 7.2 Hz, 3H, CH_3_), 1.50–1.56 (sx, *J* = 7.2 Hz, 2H, CH_2_CH_3_), 1.73–1.78 (p, *J* = 7.2 Hz, 2H, CH_2_CH_2_CH_2_), 2.29 (s, 3H, NCH_3_), 2.32–2.35 (m, 2H, CH_2_N), 2.42–2.45 (t, *J* = 7.2 Hz, 2H, CH_2_NCH_3_), 2.52–2.54 (m, 4H, CH_2piperazine_), 2.59–2.61 (m, 2H, NCH_2_CH_2_Ph), 2.64–2.66 (t, *J* = 7.2 Hz, 2H, CH_2thiazole_), 2.74–2.77 (m, 2H, CH_2_Ph), 3.43–3.45 (m, 4H, CH_2piperazine_), 6.82 (s, 1H, H_thiazole_), 7.18–7.19 (m, 3H, H), 7.26–7.28 (m, 2H, H) ppm; ^13^C-NMR (75 MHz, CDCl_3_): δ = 11.84, 19.95, 24.77, 28.93, 33.81, 42.11, 48.40, 52.37, 56.50, 59.52, 60.55, 125.88, 127.41, 128.28, 128.65, 135.47, 140.57, 170.63 ppm; IR (nujol): 3027, 2938, 2874, 2786, 1519, 1453, 1376, 1280, 1231, 1168, 1145, 1004, 911, 845, 737, 699 cm^−1^. Anal. calcd for C_22_H_37_ Br_3_N_4_S × H_2_O (M = 647.38): C 40.81, H 6.07, N 8.65, found: C 41.06, H 6.13, N 8.48.

*N-Methyl-N-(3-phenylpropyl)-3-[2-(4-n-propylpropylpiperazin-1-yl)-1,3-thiazol-5-yl]-propan-1-amine* (**3c**). A sticky oil; yield 52.0% (0.22 g); R_f_ = 0.24 (CHCl_3_/CH_3_OH 6:1); m.p._trihydrobromide monohydrate_ = 252–254 °C; ^1^H-NMR (300 MHz, CDCl_3_): δ = 1.51–1.55 (sx, *J* = 7.6 Hz, 2H, CCH_2_C); 1.73–1.80 (m, 2H, CCH_2_C); 2.21 (s, 3H, CH_3_); 2.33–2.38 (m, 6H CH_2_N, CH_2_Ph); 2.51–2.54 (m, 4H CH_2piperazine_); 2.61–2.64 (t, *J* = 7.9 Hz, 2H, CH_2thiazole_); 2.66–2.69 (t, *J* = 7.3 Hz, 2H, CH_2_N), 3.43–3.45 (m, 4H, CH_2piperaz_), 6.84 (s, 1H, H_thiazole_); 7.17–7.18 (m, 3H, H_arom_), 7.25–7.28 (m, 2H, H_arom_); ^13^C-NMR (CDCl_3_): δ = 12.07, 20.17, 25.08, 29.11, 29.11, 29.16, 33.85, 42.29, 52.59, 56.89, 57.45, 60.77, 125.88, 127.66, 128.46, 128.56, 135.63, 142.48, 170.84. IR (nujol): 3025, 2939, 2874, 2788, 1519, 1453, 1376, 1279, 1231, 1168, 1135, 1032, 1004, 911, 843, 802, 751, 699 cm^−1^. Anal. calcd for C_23_H_39_Br_3_N_4_S (M = 643.385): C 42.93, H 6.11, N 8.71, found: C 42.75, H 5.85, N 8.76.

*N-Benzyl-N-methyl-4-[2-(4-n-propylpiperazin-1-yl)-1,3-thiazol-5-yl]butan-1-amine* (**3d**). A sticky oil; yield 81.0% (0.313 g); R_f_ = 0.38 (CHCl_3_/CH_3_OH/NH_3aq_ 100:10:1); m.p._trihydrobromide dihydrate_ = 196–197 °C; ^1^H-NMR (300 MHz, CDCl_3_): δ = 0.90–0.93 (t, *J* = 7.4 Hz, 3H, CH_3_), 1.48–1.63 (m, 6H, CH_2_C), 2.17 (s, 3H, NCH_3_), 2.29–2.38 (m, 4H, CH_2_N), 2.49–2.53 (m, 4H, CH_2piperazine_); 2.62–2.64 (t, *J* = 7.0 Hz, 2H, CH_2thiazole_), 3.43–3.44 (m, 4H, CH_2piperazine_), 3.46 (s, 2H, CH_2_Ph 6.82), 6.81 (s, 1H, H_thiazole_); 7.23–7.31 (m, 5H) ppm; ^13^C-NMR (75 MHz, CDCl_3_): δ = 12.11, 20.13, 26.78, 27.08, 29.19, 42.37, 48.58, 52.56, 57.64, 60.84, 62.54, 127.00, 127.89, 128.31, 128.43, 135.51, 139.41, 170.74 ppm; IR (nujol): 3027, 2935, 2874, 2785, 1519, 1453, 1376, 1278, 1231, 1168, 1145, 1037, 1004, 911, 843, 802, 738, 699 cm^−1^. Anal. calcd for C_22_H_37_ Br_3_N_4_S × 2H_2_O (M = 665.392): C 39.71, H 5.91, N 8.43, found: C 40.03, H 5.94, N 8.46.

*N-Methyl-N-(2-phenylethyl)-4-[2-(4-n-propylpiperazin-1-yl)-1,3-thiazol-5-yl]butan-1-amine* (**3e**). A sticky oil yield 42.0% (0.168 g); R_f_ = 0.16(CHCl_3_/CH_3_OH/NH_3aq_ 200:10:1); mp_trihydrobromide monohydrate_ = 196–197 °C; ^1^H-NMR (300 MHz, CDCl_3_): δ = 0.81–0.86 (t, *J* = 7.2 Hz, 3H, CH_3_), 1.40–1.50 (m, 6H, CH_2_C), 2.21–2.24 (m, 5H, NCH_3_, CH_2_N_propyl_), 2.42–2.45 (m, 4H, CH_2piperazine_), 2.51–2.57 (m, 4H, CH_2_N), 3.34–3.37 (m, 4H, CH_2piperazine_) 6.75 (s, 1H, H_thiazole_), 7.09–7.22 (m, 5H, H) ppm; ^13^C-NMR (75 MHz, CDCl_3_): δ = 12.08, 20.10, 26.64, 27.11, 29.31, 33.84, 42.25, 48.43, 52.44, 57.25, 59.61, 60.61, 125.89, 127.51, 128.27, 128.60, 135.28, 140.33, 170.44 ppm; IR (nujol): 3027, 2935, 2875, 2785, 1519, 1453, 1376, 1278, 1231, 1168, 1145, 1037, 1004, 911, 843, 802, 738, 699 cm^−1^. Anal. calcd forC_23_H_39_Br_3_N_4_S × H_2_O (M = 661.402): C 41.76, H 6.24, N 8.42, found: C 41.82, H 6.27, N 8.51.

*N-Methyl-N-(3-phenylpropyl)-4-[2-(4-n-propylpiperazin-1-yl)-1,3-thiazol-5-yl]butan-1-amine* (**3f**). A sticky oil yield 24.0% (0.098 g); R_f_ = 0.18 (CHCl_3_/CH_3_OH/NH_3aq_ 100:10:1); mp_trihydrobromide_ = 208–210 °C; ^1^H-NMR (300 MHz, CDCl_3_): δ = 0.89–0.94 (t, *J* = 7.2 Hz, 3H, CH_3_), 1.49–1.59 (m, 6H, CH_2_C), 1.79–1.82 (m, 2H, NCH_2_CH_2_CH_2_Ph), 2.20 (s, 3H, NCH_3_), 2.31–2.38 (m, 6H, CH_2_N_propyl_, CH_2_NCH_3_), 2.51–2.54 (m, 4H, CH_2piperazine_), 2.59–2.68 (m, 4H, CH_2thiazole_, CH_2_-Ph), 3.42–3.45 (m, 4H, CH_2piperazine_), 6.83 (s, 1H, H_thiazole_), 7.14–7.29 (m, 5H, H) ppm; ^13^C-NMR (75 MHz, CDCl_3_): δ = 12.17, 20.22, 26.80, 29.21, 29.48, 33.89, 42.36, 48.55, 52.57, 57.39, 57.52, 60.74, 125.74, 127.70, 128.31, 128.40, 135.36, 142.27, 170.57 ppm; IR (nujol): 3027, 2935, 2875, 2785, 1519, 1453, 1376, 1278, 1231, 1168, 1145, 1037, 1004, 911, 843, 802, 738, 699 cm^−1^. Anal. calcd for C_24_H_41_Br_3_N_4_S (M = 657.416): C 43.84, H 6.29, N 8.52, found: C 43.51, H 6.32, N 8.53.

### 4.3. In Vitro Pharmacology

#### 4.3.1. H_3_ Antagonistic Activity for Compounds **3a**–**f**

In the first step, all obtained compounds were tested for H_3_ antagonistic effects in vitro, according to standard methods, based on electrically contracting guinea pig jejunum [33]. Male guinea pigs weighing 300–400 g were sacrificed and a portion of the small intestine, 20–50 cm proximal to the ileocaecal valve (jejunum), was removed and placed in Krebs buffer (composition (mM) NaCl 118; KCl 5.6; MgSO_4_ 1.18; CaCl_2_ 2.5; NaH_2_PO_4_ 1.28; NaHCO_3_ 25; glucose 5.5 and indomethacin (1 × 10^−6^ mol/L)). Whole jejunum segments (2 cm) were prepared and mounted between two platinum electrodes (4 mm apart) in 20 mL Krebs buffer, continuously gassed with 95% O_2_:5% CO_2_ and maintained at 37 °C. Contractions were recorded isotonically under 1.0 g tension with Hugo Sachs Hebel-Messvorsatz (Tl-2)/HF-modem (Hugo Sachs Elektronik, Hugstetten, Germany) connected to a pen recorder. The equilibration took place for one hour with washings every 10 min. The muscle segments were then stimulated at a maximum between 15 and 20 V, continuously at a frequency of 0.1 Hz for a duration of 0.5 ms, with rectangular-wave electrical pulses, delivered by a Grass Stimulator S-88 (Grass Instruments Co., Quincy, MA, USA). After 30 min of stimulation, pyrilamine (1 × 10^−5^ mol/L concentration in organ bath) was added, followed by (*R*)-*α*-methylhistamine five minutes later, and then cumulative concentration-response curves (half-log increments) of (*R*)-*α*-methylhistamine, H_3_-agonist, were recorded until no further change in response was found. Five minutes before adding the tested compounds, pyrilamine (1 × 10^−5^ mol/L concentration in an organ bath) was added, after another 20 min cumulative concentration-response curves (half-log increments) of (*R*)-*α*-methylhistamine, an H_3_-agonist, were recorded until no further change in response was found. Statistical analysis was carried out with the Students’ *t*-test. In all tests, *p* < 0.05 was considered statistically significant. The potency of an antagonist is expressed by its pA_2_ value, calculated from the Schild [33] regression analysis where at least three concentrations were used. The pA_2_ values were compared with the potency of thioperamide.

#### 4.3.2. H_1_ Antagonistic Activity for Compounds **3a**,**d**

Selected final compounds were tested for H_1_ antagonistic effects in vitro, following standard methods, using the guinea pig ileum [34]. The donors were male guinea pigs (300–400 g) as mentioned above. The excised ileum was placed in phosphate buffer at room temperature (pH 7.4) containing (mM) NaCl (136.9); KCl (2.68); NaHPO_4_ (7.19). The intraluminal content was flushed, and segments about 2 cm in lenght were cut and mounted for isotonic contractions in water mixed with 20 mL organ baths filled with oxygenated (O_2_:CO_2_ = 95:5, *v*/*v*) Krebs buffer containing (mM) NaCl (117.5); KCl (5.6); MgSO_4_ (1.18); CaCl_2_ (2.5); NaH_2_PO_4_ (1.28); NaHCO_3_ (25); glucose (5.5) and indomethacin (1 × 10^−6^ mol/L) at 37 °C under a constant load of 0.5 g. After a 30 min equilibration period with washings every 10 min, a submaximal priming dose of histamine (1 mM) was given and washed out (standard washing procedure: 3 changes of buffer during 30 min). After washing out, the antagonistic activity of given compounds was measured by recording a Concentration Response Curve (CRC) for histamine in the presence of the tested compounds **3a** and **3d**, which were added 5 min before histamine. This procedure was repeated with higher concentrations of the compounds. The antagonism was of a competitive nature causing a parallel shift of the CRC. The pA_2_-values were calculated according to Arunlakshana and Schild [34]. The pA_2_ values were compared with the affinity of pyrilamine.

#### 4.3.3. Antagonist Binding to the Rat rH_3_R and Human hH_3_R

##### Cell Culture and Transfection

Human Embryonic Kidney cells (HEK293T) were cultured in DMEM supplemented with 10% Fetal Bovine Serum and 100 IU·mL^−1^ penicillin and 100 μg·mL^−1^ streptomycins at 37 °C and 5% CO_2_. The day prior transfection two million cells were seeded in 10 cm dishes. Approximately four million cells were transfected by the polyethyleneimine (PEI) method with 5 μg of cDNA in a ratio of 1:4 (DNA:PEI). Briefly, 0.5 μg of pcDNA3-rH_3_R or pcDNA3.1-hH_3_R and 4.5 μg of empty plasmid (pcDNA3.1) were mixed with 20 μg of 25 kDa linear PEI in 500 μL of 150 mM NaCl and incubated for 30 min at 22 °C. Meanwhile, medium from 10 cm dishes was replaced with fresh culture medium and the transfection mix was subsequently added drop-wise to the cells and incubated for 48 h at 37 °C and 5% CO_2_.

##### Crude Membrane Extracts

Forty-eight hours after transfection cells were washed with ice-cold phosphate buffered saline (PBS) scrapped and the homogenate centrifuged for 10 min at ~2000 *g*, 4 °C. The supernatant was aspirated and cell pellets were resuspended in 1 mL ice-cold PBS and centrifuged again under same conditions, the supernatant aspirated and the membranes stored at −20 °C until further use.

##### [^3^H]-*N*^α^-methylhistamine Binding

Frozen cell pellets were dissolved in 50 mM Tris-HCl buffer (pH 7.4), homogenized by sonication (40 Watt Labsonic 1510) for 3 to 5 s and kept on ice until use. Cell homogenates were incubated in presence of increasing concentrations of [^3^H]-NαMH (0–20 nM) in 50 mM Tris-HCl binding buffer for saturation binding. Total and non-specific binding was determined in the absence or presence of excess non-labeled thioperamide (10 μM), respectively. For the competition binding assay, homogenates were incubated with increasing concentrations of receptor ligands (10^−11^ to 10^−4^ M) and ~2.5 nM of [^3^H]-NαMH. All assays were incubated at 25 °C for two hours on a shaking table (600 rpm). The reaction was terminated by rapid filtration into 0.5% polyethyleneimine pre-soaked glass fiber C plates (GF/C Perkin Elmer) followed by three washes with ice-cold Tris-HCl buffer (pH 7.4 at 4 °C). The plates were dried for one hour at 50 °C and scintillation liquid were added to each well (25 μL). Retained radioactivity was determined by liquid scintillation counting in a Wallac Microbeta (Perkin Elmer). Protein determination for B_max_ estimation was performed with a Pierce BCA protein assay kit and measured by spectrophotometry in Power Wave X340 (Bio-Tek Instruments Inc., BioTek, Winooski, VT, USA).

##### Chemicals

Dubelcco’s Modified Eagles Medium (DMEM), Phosphate Buffered Saline (PBS) Trizma Base, polyethyleneimine solution (50%, PEI) were purchased from Sigma Aldrich. Fetal Bovine Serum (FBS, Bodinco BV, Alkmaar, The Netherlands), Penicillin/Streptomycin (streptomycin 10,000 IU·mL^−1^; penicillin and 10,000 μg·mL^−1^ (Thermo Fisher Scientific, p/a Perbio Science BVBA, Etten-Leur, Netherlands), linear 25 kDa polyethyleneimine (PEI, Polysciences, Warrington, PA, USA), [^3^H]-*N*-α-methylhistamine (specific activity 79.7 Ci/mmol, Perkin Elmer), thioperamide, (Abcam, Cambridge, MA, USA), histamine (TCI).

##### Verification of In Vivo Activity of Compound **ADS-531**

The H_3_ antagonistic activity of **ADS-531** toward the brain histamine receptors was assessed in vivo by the intravital study of feeding behavior and then, by post-mortem analyses of neurotransmitter systems in the brain tissues from the treated rats (the ethic approval number: 86/LB696/2013).

For the first stage, food intakes were measured daily during subchronic drugs administration. H_3_R antagonists show the ability to inhibit appetite. Given that the compound enters the CNS and blocks H_3_R, more histamine should be released. The released amine acting via H_1_R would exert an anorectic effect. All animal experimental procedures were performed in accordance with EU directives and local ethical regulations. Male Lewis rats 9–10 weeks of age were used. Metabolic cages (Tecniplast, Buguggiate, Italy) were used to measure feed consumption. The cages have a standardized size that allows the animal to move inside freely. Throughout the adaptive period and the experimental one, access to feed and fluid was unlimited. The illumination cycle was stable at 12 h light on, 12 h light off. Rats were individually placed in the cages four days before the onset of treatment to adapt to a new environment and conditions of housing. The consumption and excretion were recorded every morning and the monitoring continued as long as the rats stayed in metabolic cages. The treatment ran for five days from the fifth day. Subcutaneous injections of either **ADS 531** or ciproxifan used as a reference (3 mg/kg/day to n = 8 rats) in the study group and physiological saline (0.2 mL/day to n = 8 rats) in case of the control group. Records of consumption were taken as g of feed per 100 g body mass.

### 4.4. Post-Mortem Biochemical Analyses

Post-mortem brain analyses included estimation of the amine neurotransmitters concentrations as well as the activities of the degradative enzymes HNMT and MAO-A and MAO-B. The rats were sacrificed 24 h after the last injection. The brains were collected, separated into hypothalamus and the cerebral cortex according to the Glowinski and Iversen method [42], immediately frozen in liquid nitrogen and kept at −80 °C until assayed. The amine concentrations: dopamine, noradrenaline, and serotonin were measured by RIA kits and histamine by a Research ELISA kit (DIAsource ImmunoAssays S.A. Louvain-la-Neuve, Place du Levant, Belgium). The enzyme activities of histamine *N*-methyltransferase and monoamine oxidase A and B were estimated by radioisotopic assays according to Taylor and Snyder [43] and Fowler and Tipton [44] with modifications by Gomez et al. [45], respectively. For MAO-A serotonin (5-[2-^14^C]-hydroxytryptamine binoxalate) and for MAO-B β-[ethyl-1-^14^C]-phenylethylamine hydrochloride were used as substrates and respectively, clorgyline (MAO-A) and deprenyl (MAO-B) (final conc. 10^−9^ M) as the enzymes inhibitors. Protein was measured by Lowry method [46].

## Figures and Tables

**Table 1:**
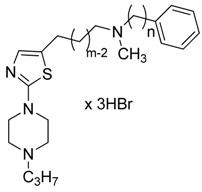
H_3_- and H_1_-antagonistic potency of compounds **2a–c and 3a–f** as tested in the in vitro test system in the guinea pig jejunum.

Cpd.	Code Cpd.	m	n	pA_2_ (SEM)		pA_2_ (SEM)	
				H_3_	N (Caviae)	H_1_	N (Caviae)
**2a** [31]	**ADS-529**	2	1	7.76 (0.06)	18 (5)	NA	
**2b** [32]	**ADS-530**	2	2	7.61 (0.06)	9 (3)	NA	
**2c** [31]	**ADS-531**	2	3	8.27 (0.05)	20 (6)	NA	
**3a**	**ADS-562**	3	1	8.38 (0.02)	15 (5)	5.5 (0.11)	9 (3)
**3b**	**ADS-563**	3	2	7.81 (0.10)	12 (4)	NT	
**3c**	**ADS-564**	3	3	7.46 (0.11)	12 (4)	NT	
**3d**	**ADS-565**	4	1	7.95 (0.10)	12 (4)	6.25 (0.13)	9 (3)
**3e**	**ADS-566**	4	2	7.97 (0.03)	12 (4)	NT	
**3f**	**ADS-567**	4	3	7.91 (0.12)	12 (4)	NT	

Thioperamide—pA_2_ H_3_ = 8.47, (SEM; 0.26); N (caviae)—36 (12); Pyrilamine—pA_2_ H_1_ = 8.66, (SEM; 0.03); N (caviae)—18 (6); SEM—standard error of the mean; N—number of different animal preparations; (caviae)—number of animals; NA, inactive; NT, not tested.

**Table 2:** The effect of subchronic administration of **ADS-531** (s.c. 3 mg/kg/daily for five days) or ciproxifan (s.c. 3 mg/kg/daily for 5 days) on cerebral MAOs and HNMT activities.

Group	MAO-A	MAO-B	HNMT	
	pmol/min/mg protein		pmol/min/mg protein	
	CTX	CTX	CTX	HTH
**ADS-531**	1625 ± 49.99	1044 ± 38.91	48.19 ± 0.47	37.58 ± 2.42
**Ciproxifan**	1654 ± 31.42	1049 ± 56.16	46.90 ± 3.59	44.68 ± 5.50
**Control**	1506 ± 29.42	1054 ± 19.31	45.97 ± 1.28	34.33 ± 3.18

The values are given as means ± SEM for seven to eight rats; The drugs were administered subcutaneously at a dose of 3 mg/kg of body mass for five consecutive days; MAO—monoamine oxidase, HNMT—histamine *N*-methyltransferase, CTX—cerebral cortex, HTH—hypothalamus.

## Supplementary Materials

The following are available online.
