# Supplementary material for: Non-Imidazole Histamine H3 Ligands. Part VII. Synthesis, In Vitro and In Vivo Characterization of 5-Substituted-2-thiazol-4-n-propylpiperazines
Source: Molecules. 2018 Feb 3;23(2):326. doi: 10.3390/molecules23020326 (PMC6017745; doi:10.3390/molecules23020326)
Supplement: Supplementary file 1 [file molecules-23-00326-s001.zip › KW Appendix A to the revised manuscript 256480.pdf]

“Non-imidazole histamine H_3 ligands. Part VII. Synthesis, in vitro and in vivo characterization of 5-substituted-2-thiazol-4-n-propylpiperazines”

Appendix A

Supplementary data

^{1}H and ^{13}C spectra for final compounds 3a-f

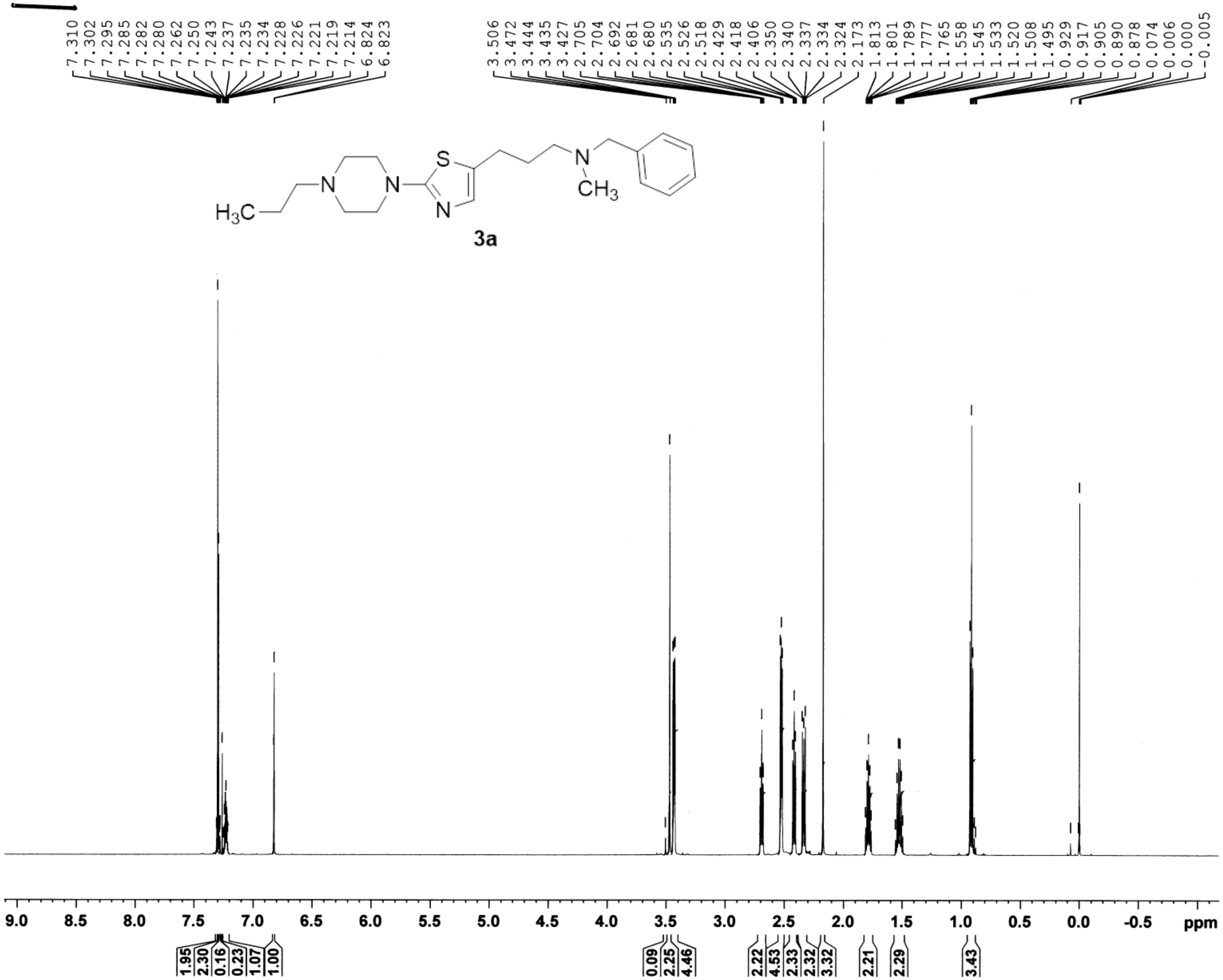

NAME	rga 1011
EXPNO	11
PROCNO	1
Date_	20110410
Time	0.05
INSTRUM	spect
PROBHD	5 mm PABBO BB-
PULPROG	zg30
TD	65536
SOLVENT	CDCl3
NS	16
DS	2
SWH	12335.526 Hz
FIDRES	0.188225 Hz
AQ	2.6564426 sec
RG	50.8
DW	40.533 usec
DE	8.50 usec
TE	303.1 K
D1	1.00000000 sec
TD0	1

======== CHANNEL f1 ========

NUC1	1H
P1	9.40 usec
PL1	-3.00 dB
PL1W	30.57242203 W
SFO1	600.2637069 MHz
SI	32768
SF	600.2600167 MHz
WDW	no
SSB	0
LB	0.00 Hz
GB	0
PC	1.00

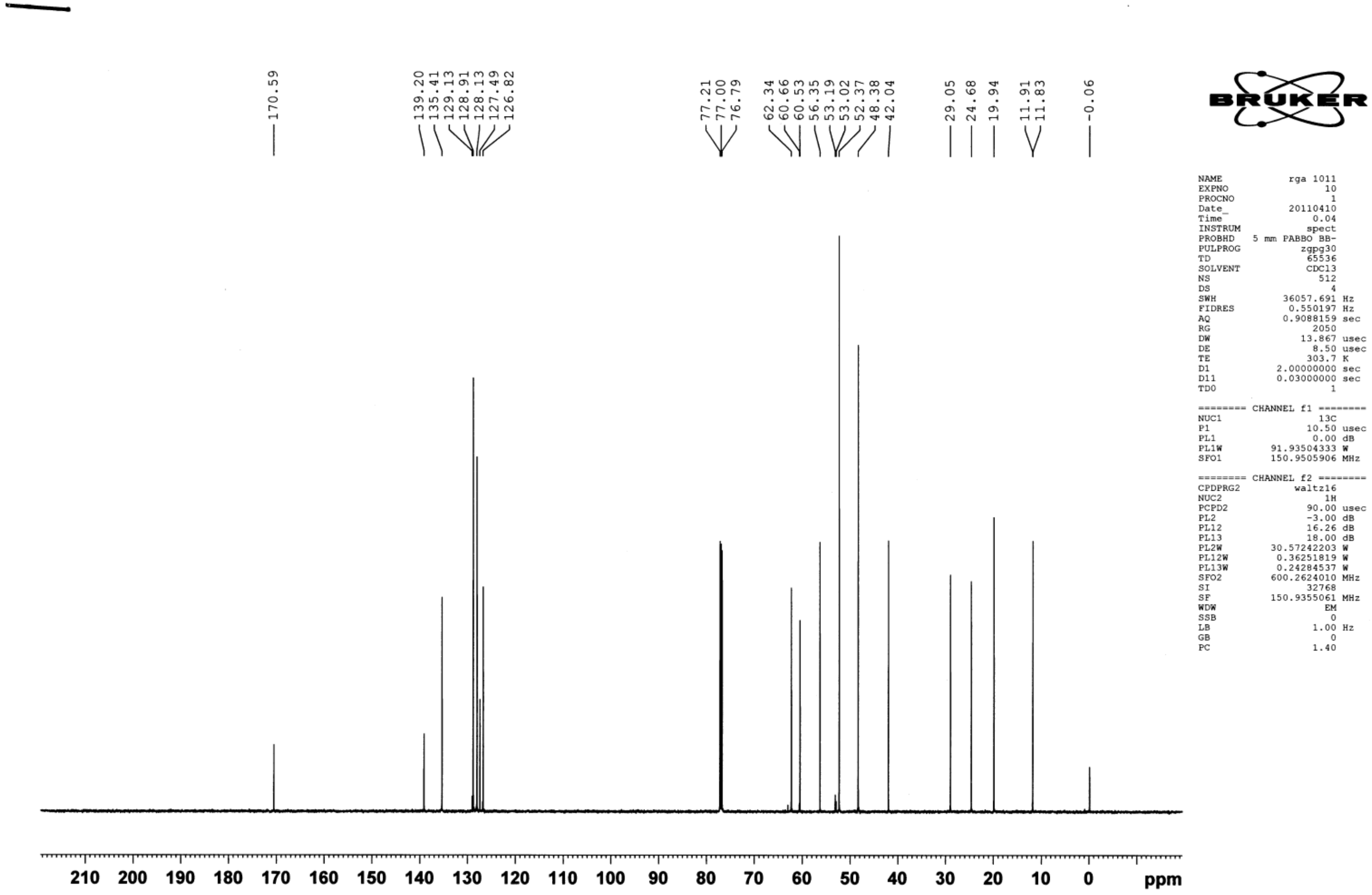
170.59
139.20
135.41
129.13
128.91
128.13
127.49
126.82
77.21
77.00
76.79
62.34
60.66
60.53
56.35
53.19
53.02
52.37
48.38
42.04
29.05
24.68
19.94
11.91
11.83
-0.06
210 200 190 180 170 160 150 140 130 120 110 100 90 80 70 60 50 40 30 20 10 0 ppm
BRUKER
NAME rga 1011
EXPNO 10
PROCNO 1
Date_ 20110410
Time 0.04
INSTRUM spect
PROBHD 5 mm PABBO BB-
PULPROG zgpg30
TD 65536
SOLVENT CDCl3
NS 512
DS 4
SWH 36057.691 Hz
FIDRES 0.550197 Hz
AQ 0.9088159 sec
RG 2050
DW 13.867 usec
DE 8.50 usec
TE 303.7 K
D1 2.00000000 sec
D11 0.03000000 sec
TD0 1
======== CHANNEL f1 ========
NUC1 13C
P1 10.50 usec
PL1 0.00 dB
PL1W 91.93504333 W
SFO1 150.9505906 MHz
======== CHANNEL f2 ========
CPDPRG2 waltz16
NUC2 1H
PCPD2 90.00 usec
PL2 -3.00 dB
PL12 16.26 dB
PL13 18.00 dB
PL2W 30.57242203 W
PL12W 0.36251819 W
PL13W 0.24284537 W
SFO2 600.2624010 MHz
SI 32768
SF 150.9355061 MHz
WDW EM
SSB 0
LB 1.00 Hz
GB 0
PC 1.40

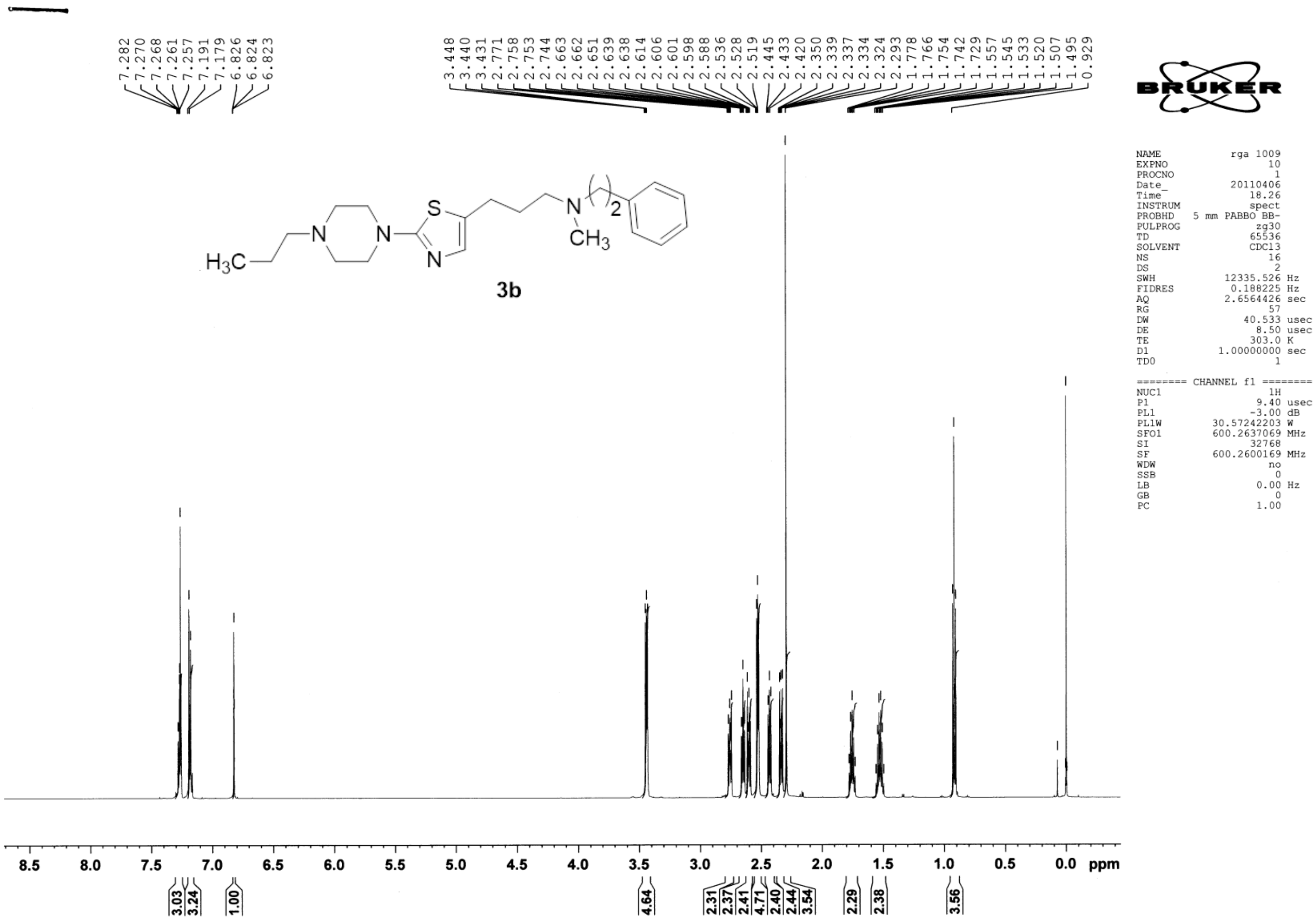
7.282
7.270
7.268
7.261
7.257
7.191
7.179
6.826
6.824
6.823
3.448
3.440
3.431
2.771
2.758
2.753
2.744
2.663
2.662
2.651
2.639
2.638
2.614
2.606
2.601
2.598
2.588
2.536
2.528
2.519
2.445
2.433
2.420
2.350
2.339
2.337
2.334
2.324
2.293
1.778
1.766
1.754
1.742
1.729
1.557
1.545
1.533
1.520
1.507
1.495
0.929
S
N
N
N
H3C
N
2
CH3
3b
NAME rga 1009
EXPNO 10
PROCNO 1
Date_ 20110406
Time 18.26
INSTRUM spect
PROBHD 5 mm PABBO BB-
PULPROG zg30
TD 65536
SOLVENT CDCl3
NS 16
DS 2
SWH 12335.526 Hz
FIDRES 0.188225 Hz
AQ 2.6564426 sec
RG 57
DW 40.533 usec
DE 8.50 usec
TE 303.0 K
D1 1.00000000 sec
TD0 1
======== CHANNEL f1 ========
NUC1 1H
P1 9.40 usec
PL1 -3.00 dB
PL1W 30.57242203 W
SFO1 600.2637069 MHz
SI 32768
SF 600.2600169 MHz
WDW no
SSB 0
LB 0.00 Hz
GB 0
PC 1.00
8.5 8.0 7.5 7.0 6.5 6.0 5.5 5.0 4.5 4.0 3.5 3.0 2.5 2.0 1.5 1.0 0.5 0.0 ppm
3.03
3.24
1.00
4.64
2.31
2.37
2.41
4.71
2.40
2.44
3.54
2.29
2.38
3.56

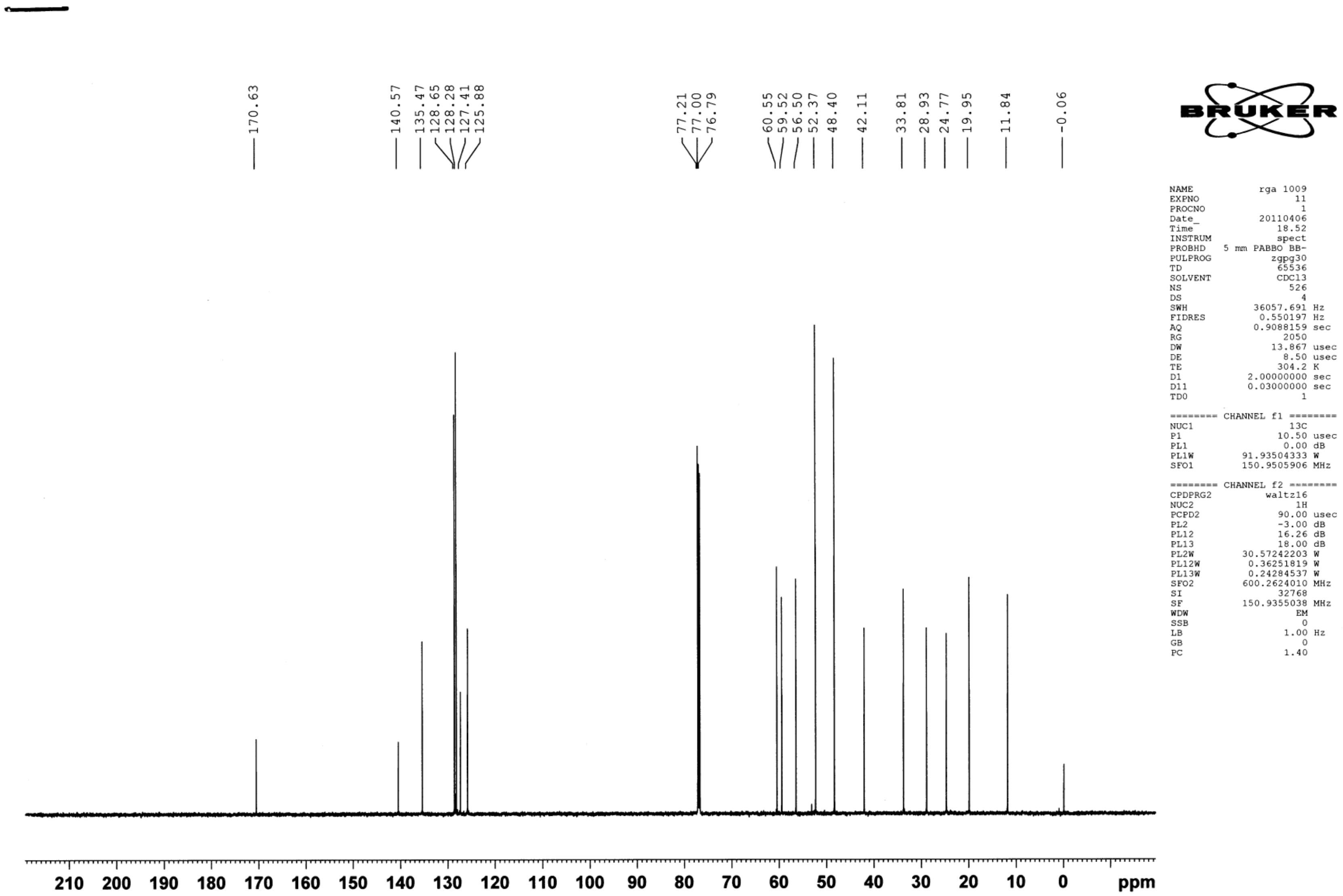
170.63
140.57
135.47
128.65
128.28
127.41
125.88
77.21
77.00
76.79
60.55
59.52
56.50
52.37
48.40
42.11
33.81
28.93
24.77
19.95
11.84
-0.06
BRUKER
NAME rga 1009
EXPNO 11
PROCNO 1
Date_ 20110406
Time 18.52
INSTRUM spect
PROBHD 5 mm PABBO BB-
PULPROG zgpg30
TD 65536
SOLVENT CDCl3
NS 526
DS 4
SWH 36057.691 Hz
FIDRES 0.550197 Hz
AQ 0.9088159 sec
RG 2050
DW 13.867 usec
DE 8.50 usec
TE 304.2 K
D1 2.00000000 sec
D11 0.03000000 sec
TD0 1
======== CHANNEL f1 ========
NUC1 13C
P1 10.50 usec
PL1 0.00 dB
PL1W 91.93504333 W
SFO1 150.9505906 MHz
======== CHANNEL f2 ========
CPDPRG2 waltz16
NUC2 1H
PCPD2 90.00 usec
PL2 -3.00 dB
PL12 16.26 dB
PL13 18.00 dB
PL2W 30.57242203 W
PL12W 0.36251819 W
PL13W 0.24284537 W
SFO2 600.2624010 MHz
SI 32768
SF 150.9355038 MHz
WDW EM
SSB 0
LB 1.00 Hz
GB 0
PC 1.40
210 200 190 180 170 160 150 140 130 120 110 100 90 80 70 60 50 40 30 20 10 0 ppm

SpinWorks 3: no title

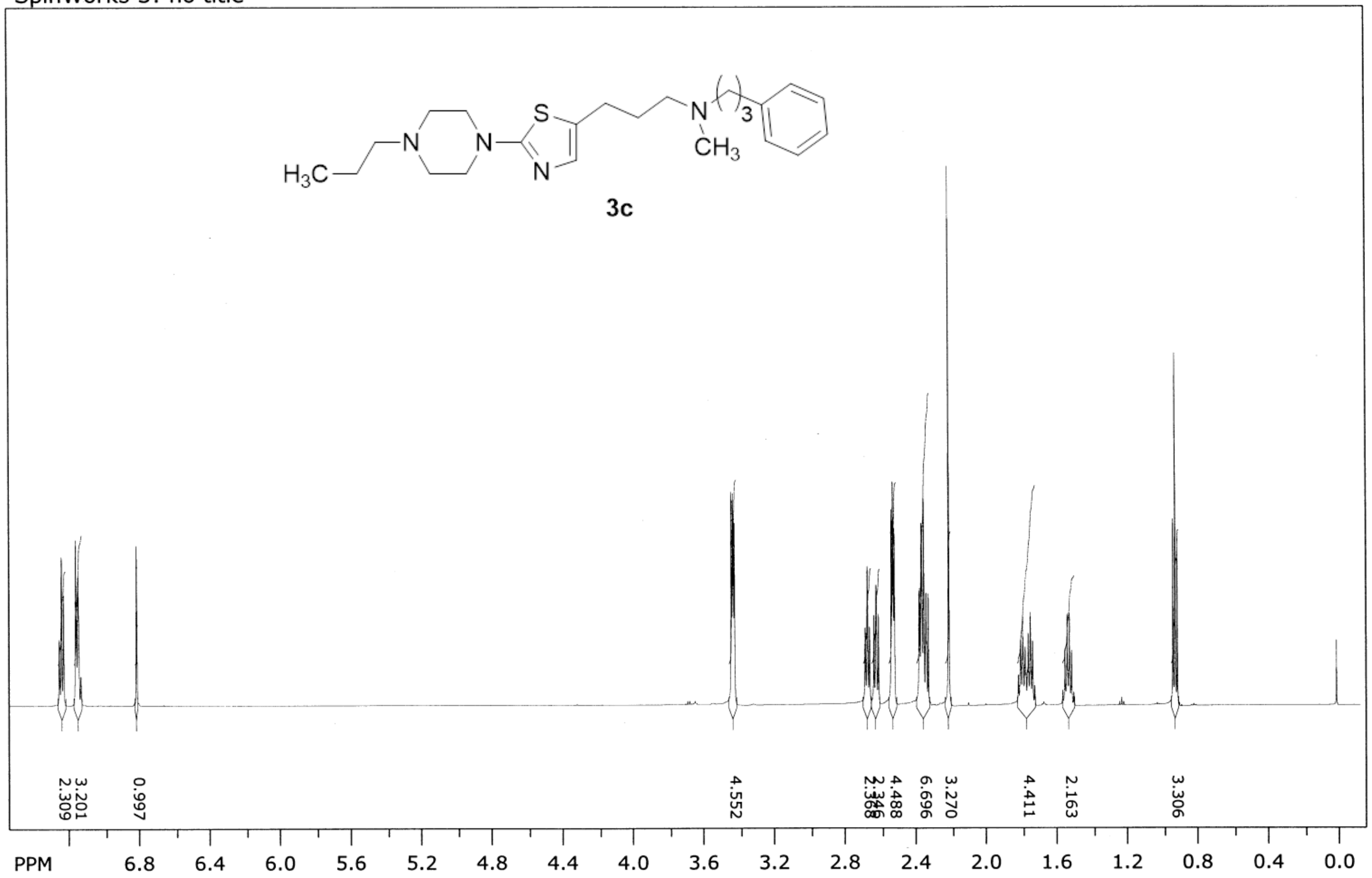

file: ...mr\Analizy bieżące\rga-1276\10\fid expt: <zg30>
transmitter freq.: 600.263707 MHz
time domain size: 65536 points
width: 12335.53 Hz = 20.5502 ppm = 0.188225 Hz/pt
number of scans: 16

freq. of 0 ppm: 600.260018 MHz
processed size: 32768 complex points
LB: 0.000 GF: 0.0000
Hz/cm: 184.934 ppm/cm: 0.30809

SpinWorks 3: no title

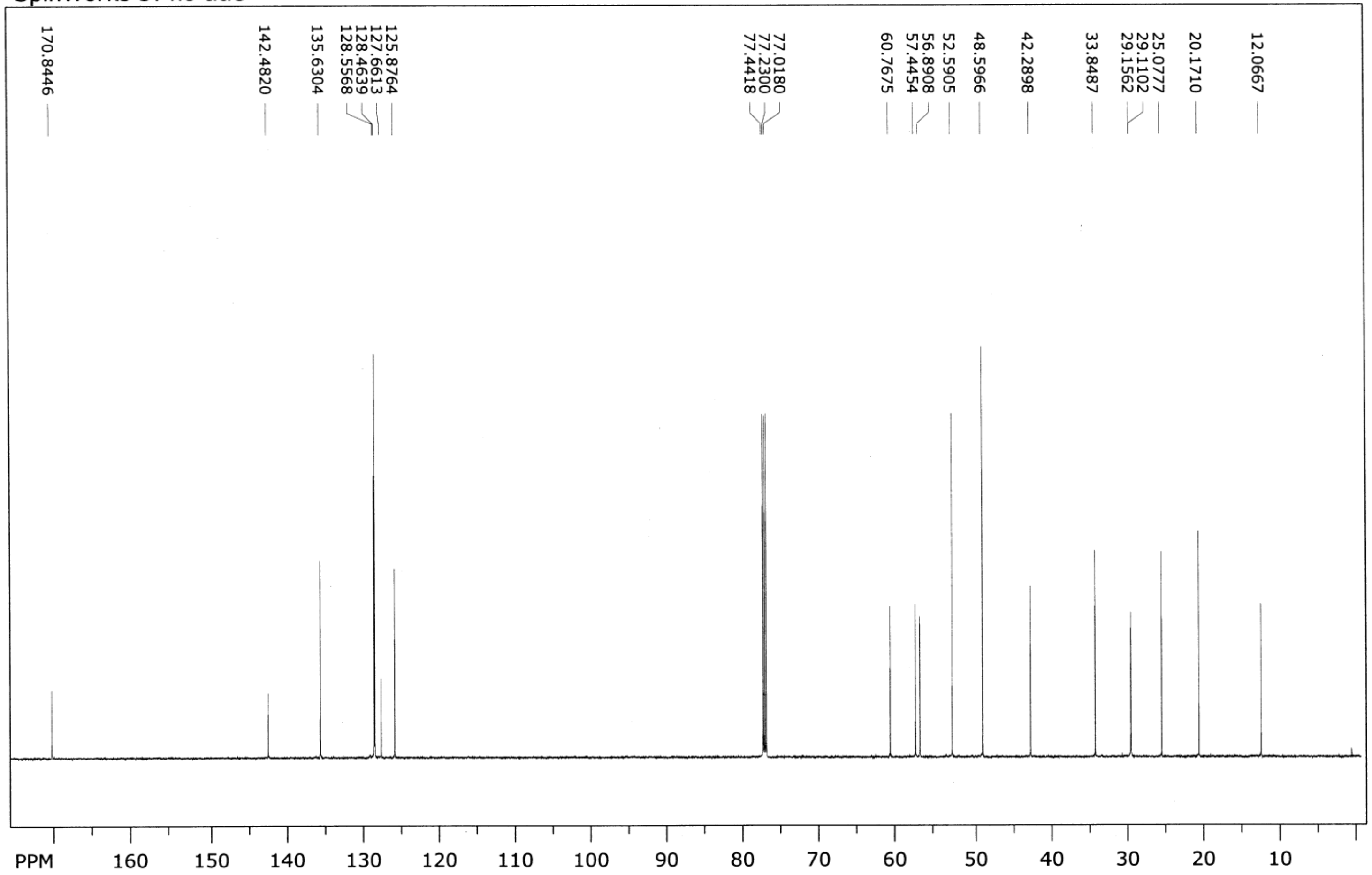

file: ...mr\Analizy bieżące\rga-1276\11\fid expt: <zgpg30>
transmitter freq.: 150.950591 MHz
time domain size: 65536 points
width: 36057.69 Hz = 238.8708 ppm = 0.550197 Hz/pt
number of scans: 1024

freq. of 0 ppm: 150.935471 MHz
processed size: 32768 complex points
LB: 0.000 GF: 0.0000
Hz/cm: 1070.734 ppm/cm: 7.09327

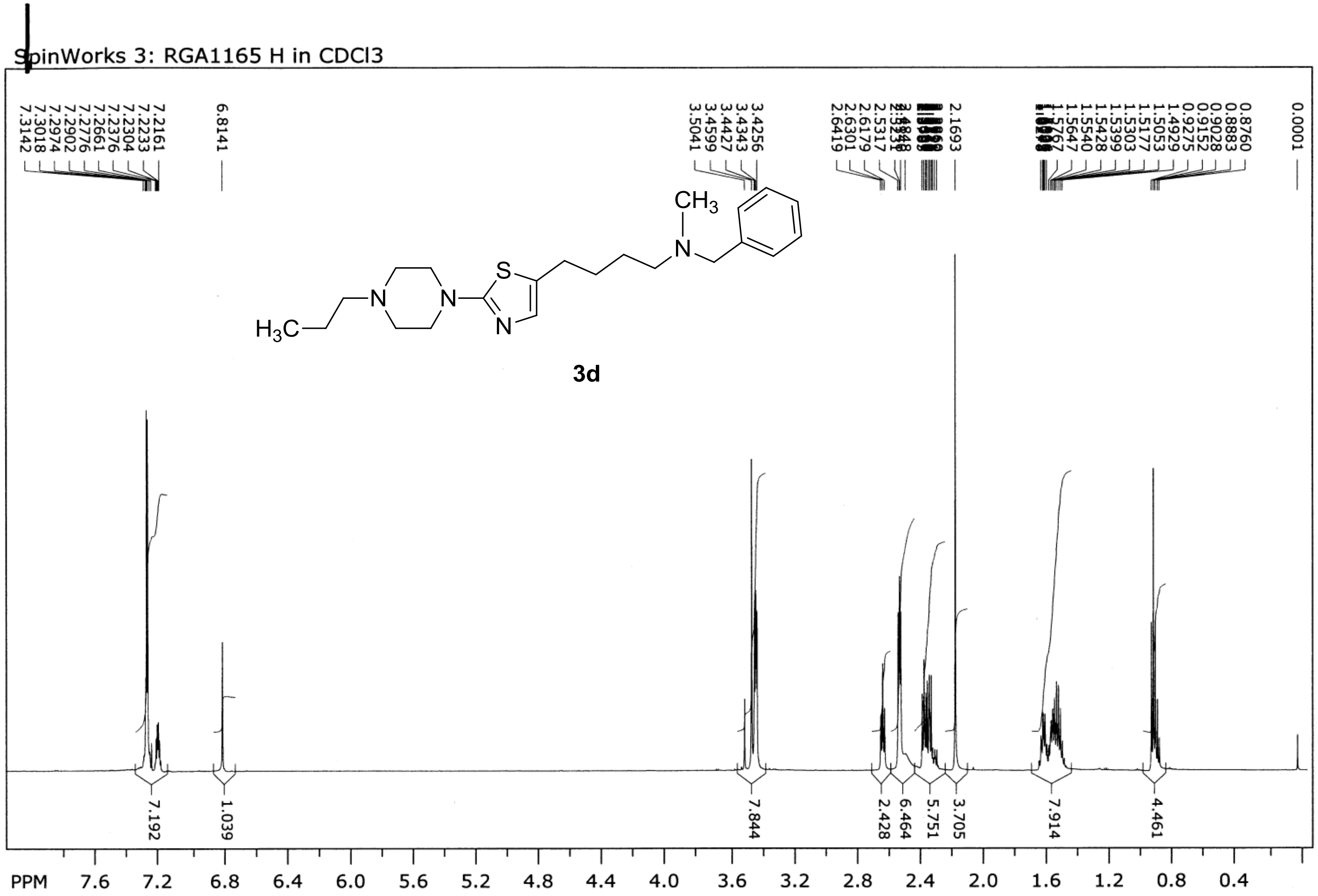

file: K:\rga-1165\11\fid expt: <zg30>
transmitter freq.: 600.263707 MHz
time domain size: 65536 points
width: 12335.53 Hz = 20.5502 ppm = 0.188225 Hz/pt
number of scans: 16

freq. of 0 ppm: 600.260014 MHz
processed size: 32768 complex points
LB: 0.300 GF: 0.0000
Hz/cm: 198.164 ppm/cm: 0.33013

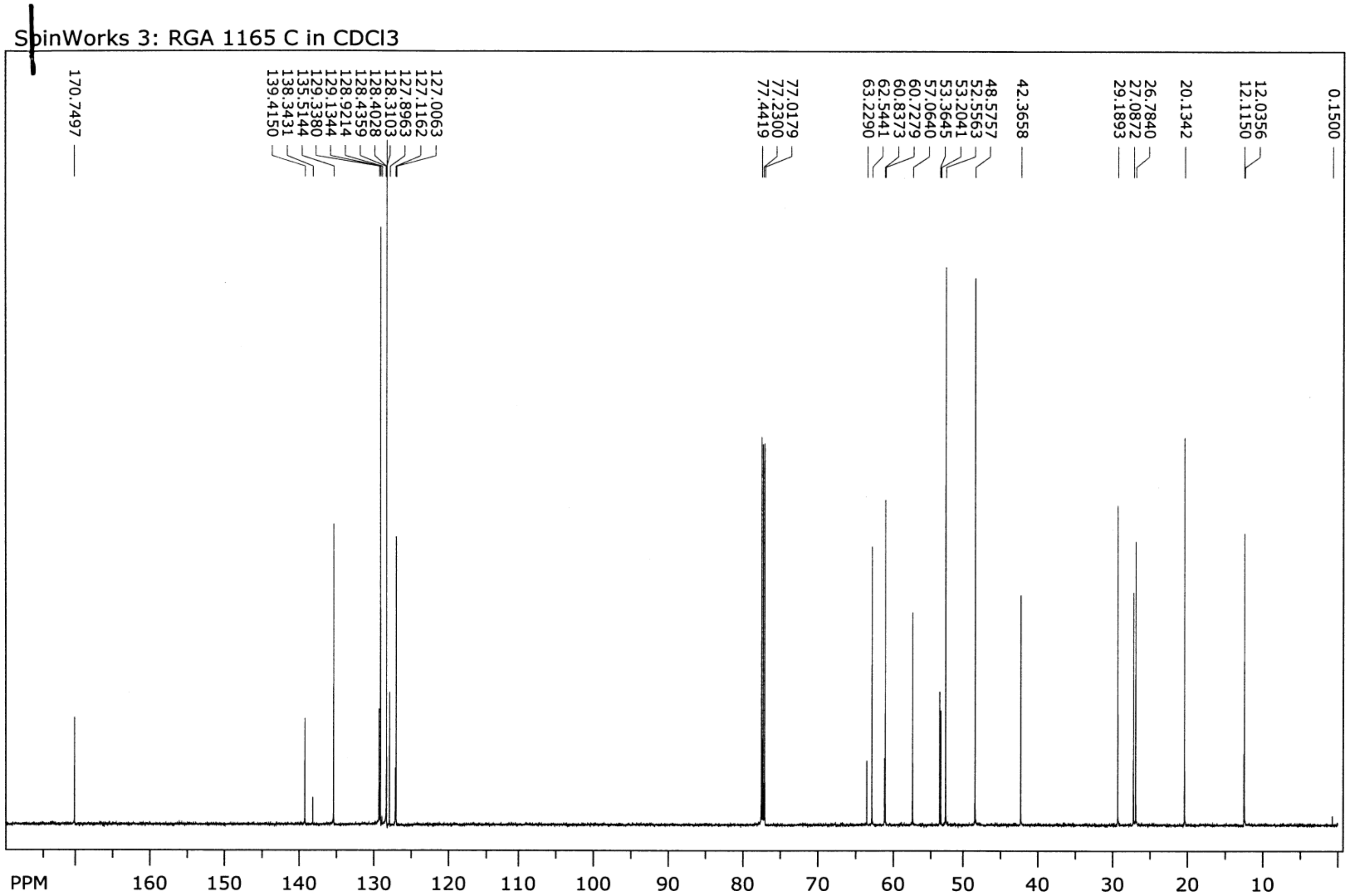

file: K:\rga-1165\10\fid expt: <zgpg30>
transmitter freq.: 150.950591 MHz
time domain size: 65536 points
width: 36057.69 Hz = 238.8708 ppm = 0.550197 Hz/pt
number of scans: 1024

freq. of 0 ppm: 150.935474 MHz
processed size: 32768 complex points
LB: 1.000 GF: 0.0000
Hz/cm: 1091.036 ppm/cm: 7.22777

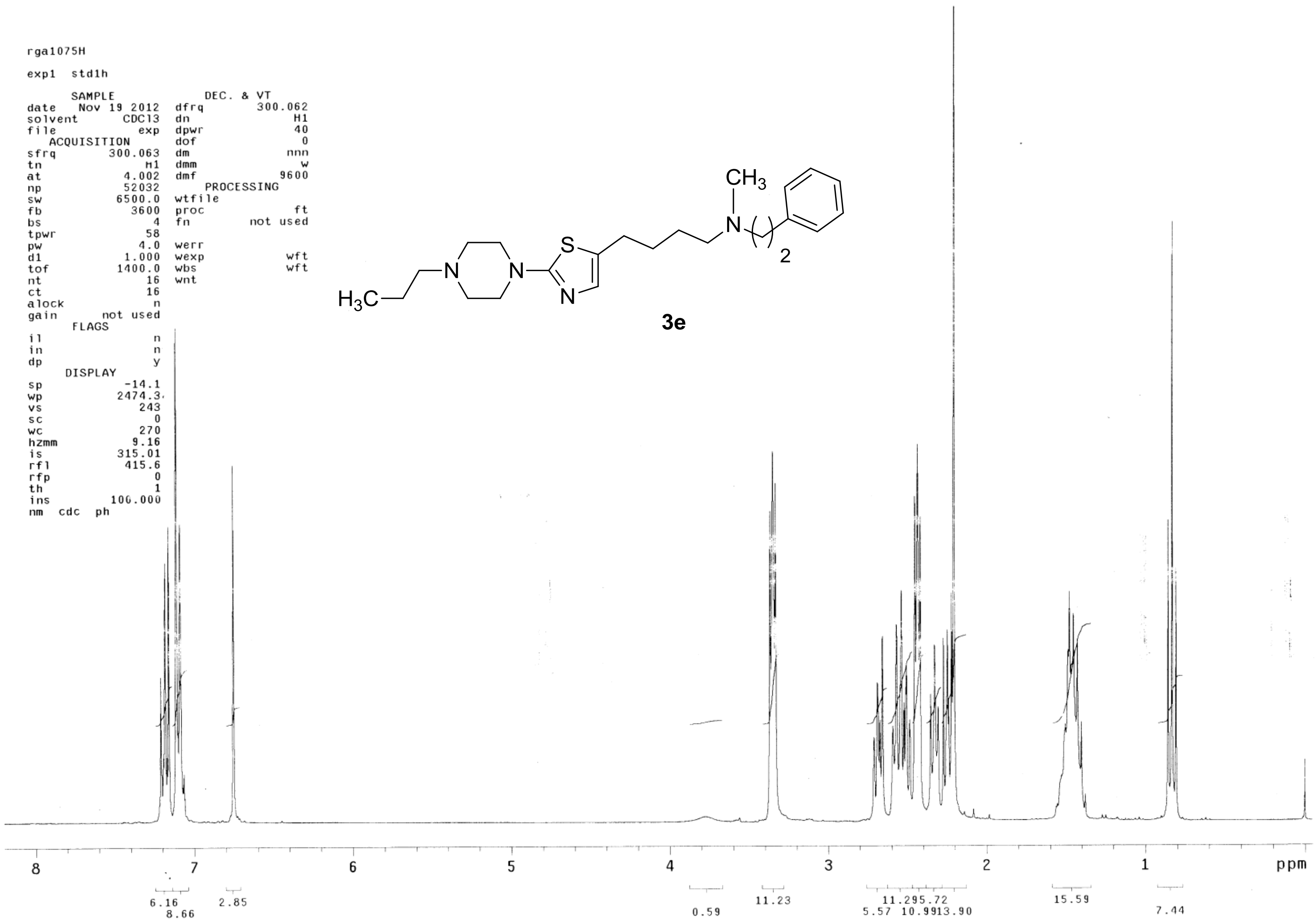
rga1075H
exp1 std1h
SAMPLE
date Nov 19 2012
solvent CDCl3
file exp
ACQUISITION
sfrq 300.063
tn H1
at 4.002
np 52032
sw 6500.0
fb 3600
bs 4
tpwr 58
pw 4.0
d1 1.000
tof 1400.0
nt 16
ct 16
alock n
gain not used
FLAGS
il n
in n
dp y
DISPLAY
sp -14.1
wp 2474.3
vs 243
sc 0
wc 270
hzmm 9.16
is 315.01
rfl 415.6
rfp 0
th 1
ins 100.000
nm cdc ph
DEC. & VT
dfrq 300.062
dn H1
dpwr 40
dof 0
dm nnn
dmm w
dmf 9600
PROCESSING
wtfile
proc ft
fn not used
werr
wexp wft
wbs wft
wnt
CH3
S
N
N
N
N
H3C
2
3e
8
7
6
5
4
3
2
1
ppm
6.16
8.66
2.85
0.59
11.23
11.29
5.72
5.57
10.99
13.90
15.59
7.44

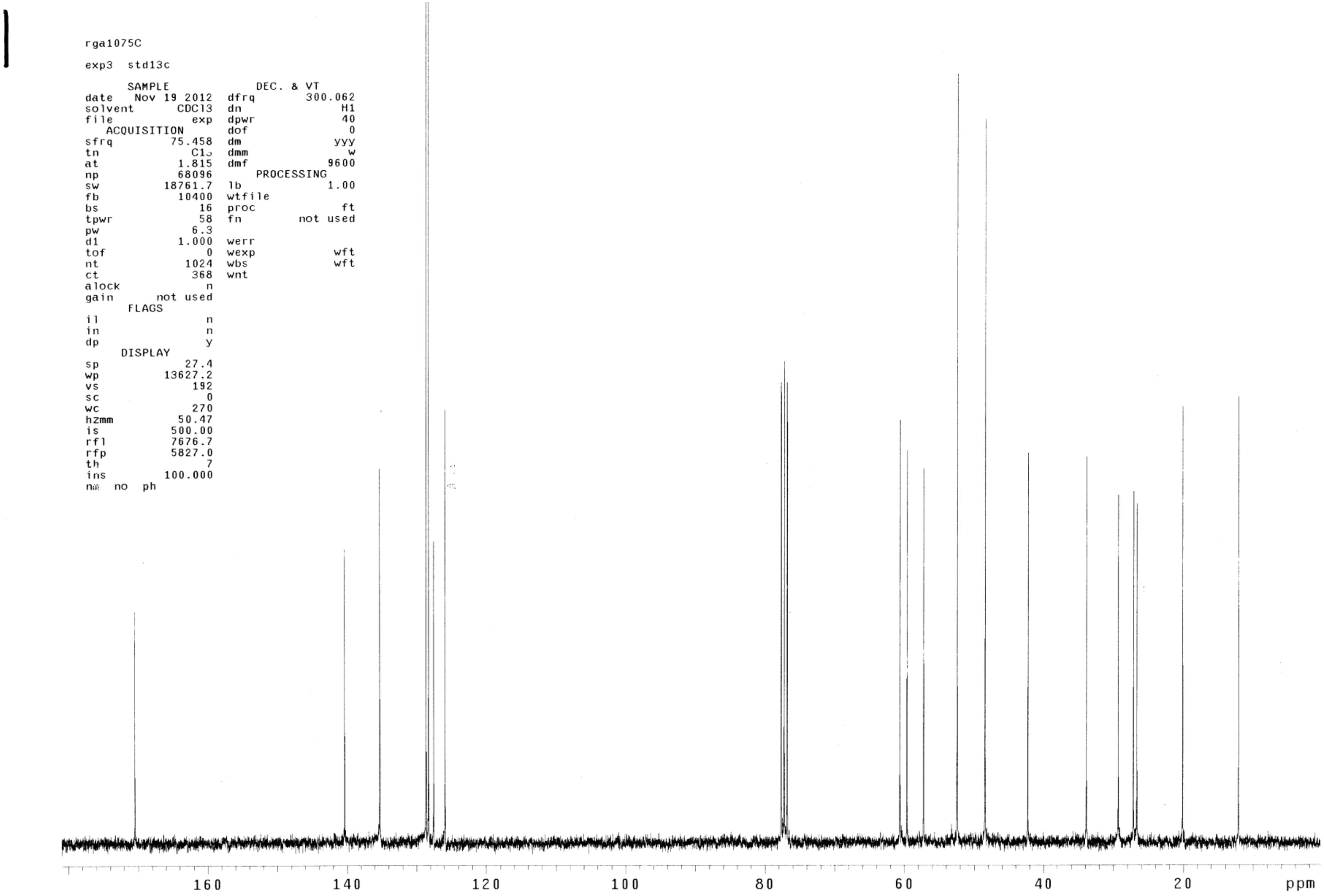
rga1075C
exp3 std13c
SAMPLE
date Nov 19 2012
solvent CDCl3
file exp
ACQUISITION
sfrq 75.458
tn C13
at 1.815
np 68096
sw 18761.7
fb 10400
bs 16
tpwr 58
pw 6.3
d1 1.000
tof 0
nt 1024
ct 368
alock n
gain not used
FLAGS
il n
in n
dp y
DISPLAY
sp 27.4
wp 13627.2
vs 192
sc 0
wc 270
hzmm 50.47
is 500.00
rfl 7676.7
rfp 5827.0
th 7
ins 100.000
nm no ph
DEC. & VT
dfrq 300.062
dn H1
dpwr 40
dof 0
dm yyy
dmm w
dmf 9600
PROCESSING
lb 1.00
wtfile
proc ft
fn not used
werr
wexp wft
wbs wft
wnt
160
140
120
100
80
60
40
20
ppm

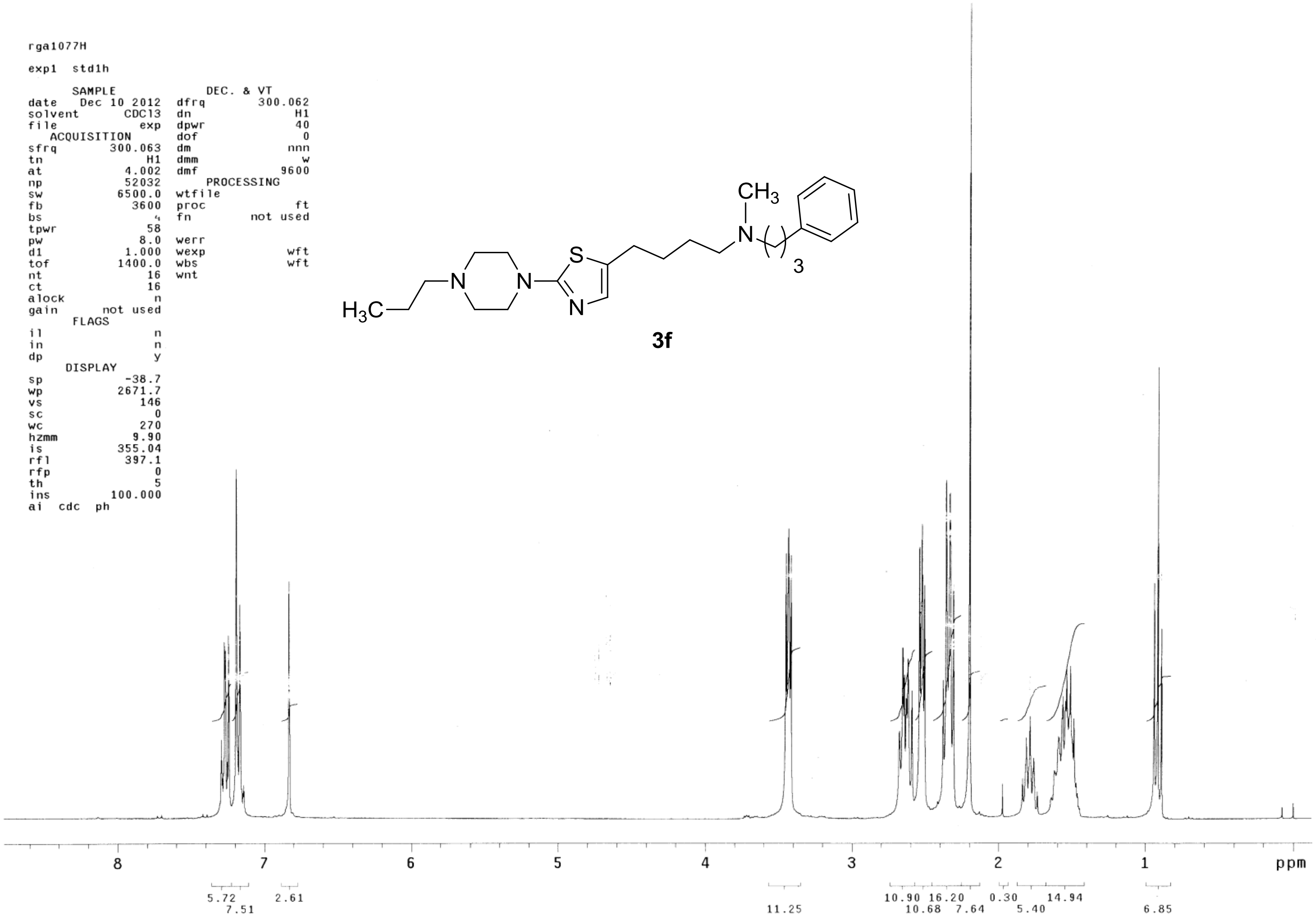
rga1077H
exp1 std1h
SAMPLE
date Dec 10 2012
solvent CDCl3
file exp
ACQUISITION
sfrq 300.063
tn H1
at 4.002
np 52032
sw 6500.0
fb 3600
bs 4
tpwr 58
pw 8.0
d1 1.000
tof 1400.0
nt 16
ct 16
alock n
gain not used
FLAGS
il n
in n
dp y
DISPLAY
sp -38.7
wp 2671.7
vs 146
sc 0
wc 270
hzmm 9.90
is 355.04
rfl 397.1
rfp 0
th 5
ins 100.000
ai cdc ph
DEC. & VT
dfrq 300.062
dn H1
dpwr 40
dof 0
dm nnn
dmm w
dmf 9600
PROCESSING
wtfile
proc ft
fn not used
werr
wexp wft
wbs wft
wnt
8 7 6 5 4 3 2 1 ppm
5.72 7.51 2.61 11.25 10.90 10.68 16.20 7.64 0.30 5.40 14.94 6.85
CH3
N
S
N
N
H3C
3

3f

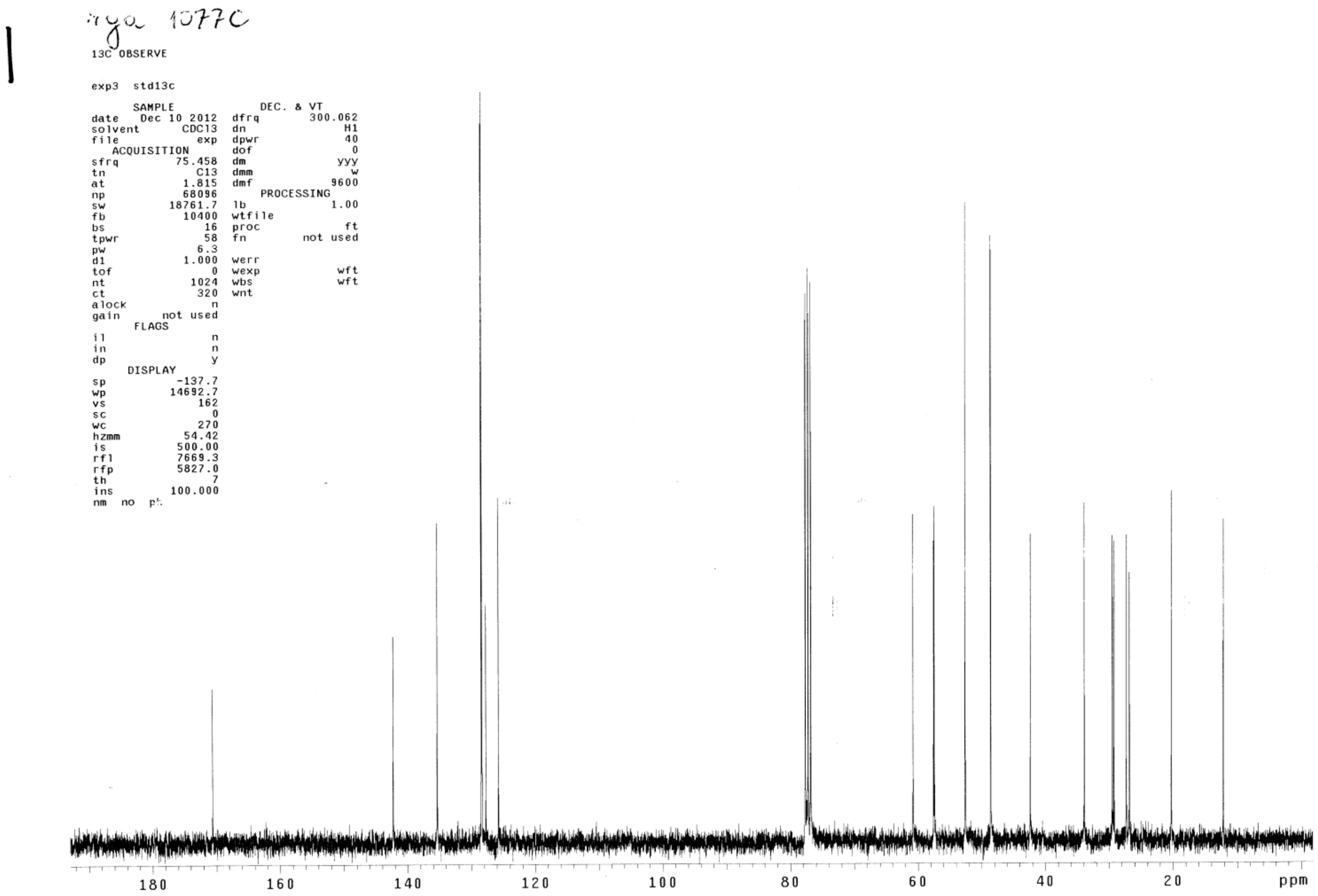
ya 1077C
13C OBSERVE
exp3 std13c
SAMPLE
date Dec 10 2012
solvent CDCl3
file exp
ACQUISITION
sfrq 75.458
tn C13
at 1.815
np 68096
sw 18761.7
fb 10400
bs 16
tpwr 58
pw 6.3
d1 1.000
tof 0
nt 1024
ct 320
alock n
gain not used
FLAGS
il n
in n
dp y
DISPLAY
sp -137.7
wp 14692.7
vs 162
sc 0
wc 270
hzmm 54.42
is 500.00
rfl 7669.3
rfp 5827.0
th 7
ins 100.000
nm no ph
DEC. & VT
dfrq 300.062
dn H1
dpwr 40
dof 0
dm yyy
dmm w
dmf 9600
PROCESSING
lb 1.00
wtfile
proc ft
fn not used
werr
wexp wft
wbs wft
wnt
180
160
140
120
100
80
60
40
20
ppm
