# Supplementary material for: Non-Imidazole Histamine H3 Ligands. Part VII. Synthesis, In Vitro and In Vivo Characterization of 5-Substituted-2-thiazol-4-n-propylpiperazines
Source: Molecules. 2018 Feb 3;23(2):326. doi: 10.3390/molecules23020326 (PMC6017745; doi:10.3390/molecules23020326)
Supplement: Supplementary file 1 [file molecules-23-00326-s001.zip › KW Appendix B to the revised manuscript 256480.pdf]

"Non-imidazole histamine H_3 ligands. Part VII. Synthesis, in vitro and in vivo characterization of 5-substituted-2-thiazol-4-n-propylpiperazines"

Appendix B

Supplementary data

Pharmacology

2.2.2 Histamine H_3 receptor affinity

Saturation of rat and human H_3 receptors

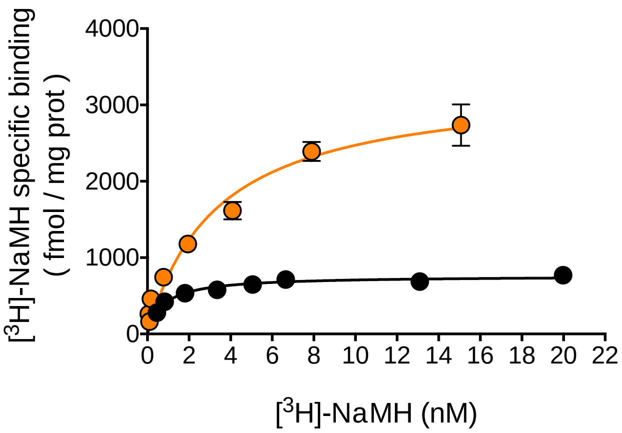

Figure S1. Saturation of rat and human H_3R.

Competition binding of H_3 receptor ligands

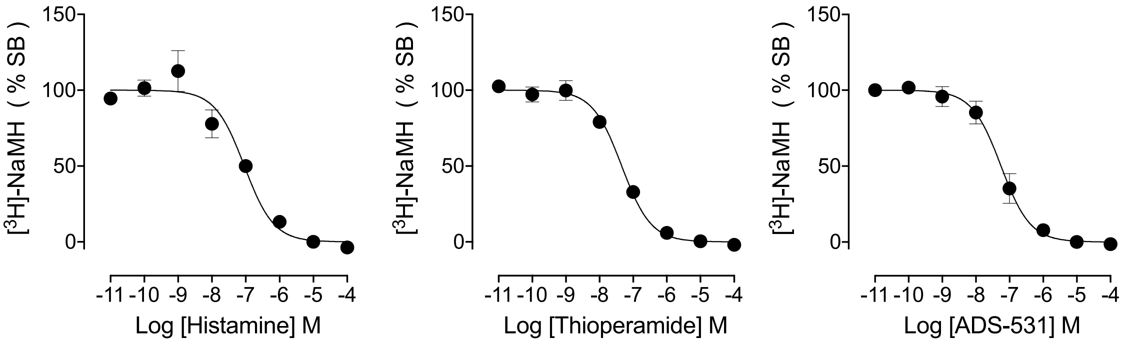

Figure S2. Competition binding of H_3R ligands on rat H_3 receptor.

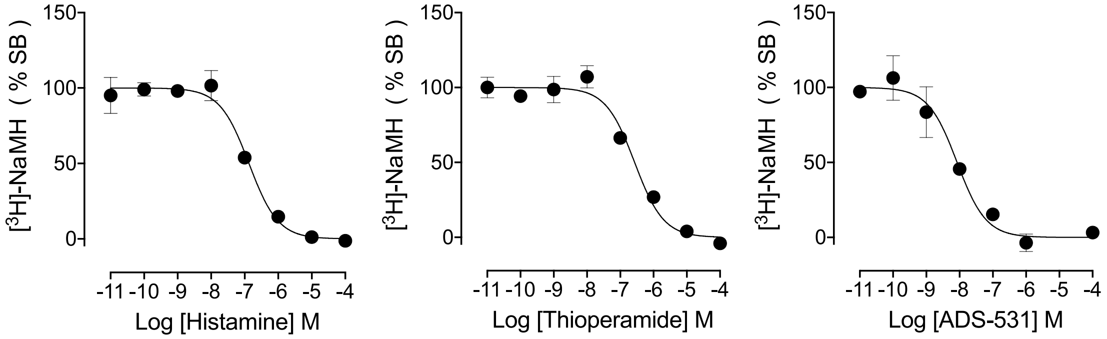

Figure S3: Competition binding of H_3R ligands on human H_3 receptor
